# Domain-specific cognitive impairment is differentially affected by Alzheimer disease tau pathologic burden and spread

**DOI:** 10.1162/imag_a_00405

**Published:** 2024-12-19

**Authors:** Stephanie Doering, Nicole S. McKay, Nayid Jana, Kaitlyn Dombrowski, Austin McCullough, Peter R. Millar, Diana A. Hobbs, Rohan Agrawal, Shaney Flores, Jorge J. Llibre-Guerra, Edward D. Huey, Beau M. Ances, Chengjie Xiong, Andrew J. Aschenbrenner, Jason Hassenstab, John C. Morris, Brian A. Gordon, Tammie L.S. Benzinger

**Affiliations:** Washington University School of Medicine, Saint Louis, MO, United States; Memory and Aging Program, Butler Hospital, Providence, RI, United States

**Keywords:** Alzheimer disease, Positron Emission Tomography (PET), Tau spread, cognitive domains, cognitive impairment

## Abstract

Tau pathology in Alzheimer disease (AD) is often evaluated in regions associated with episodic memory impairment. However, heterogeneous spreading patterns of tau are observed and correspond to impairment in different cognitive domains. We have previously developed a metric to quantify tau spread extent that is robustly sensitive to atypical spreading patterns. Here, we evaluate tau spread relative to domain-specific and general cognitive impairments during early stages of AD. In total, 529 participants with baseline tau positron emission tomography (PET) and neuropsychological testing were separated into disease-stage groups based on amyloid PET positivity and clinical status via Clinical Dementia Rating® (CDR®). General cognition was assessed using the Knight Preclinical Alzheimer Cognitive Composite (Knight PACC). Domain-specific composites were calculated for episodic memory, semantic memory, working memory, and attention/processing speed. Baseline tau burden, the average tau intensity across previously defined AD signature regions, and baseline tau spread extent, the proportion of the brain with elevated tau pathology, were quantified for each participant as Tau Index and Tau Spatial Spread, respectively. Tau burden and tau spread were evaluated relative to baseline and longitudinal cognitive performance, as well as longitudinal clinical progression. Tau burden and tau spread extent both significantly correlate with cognitive impairment in symptomatic AD. Tau burden is most strongly correlated with episodic (r = -0.37, p = 0.02) and semantic (r = -0.36, p = 0.02) memory. In contrast, tau spread extent is most strongly correlated with the Knight PACC (r = -0.37, p = 0.01) and attention/processing speed (r = -0.44, p < 0.01), especially in preclinical AD (r = -0.27, p < 0.01). Tau burden captures more variance than tau spread extent in longitudinal change in the Knight PACC, episodic memory, semantic memory, attention/processing speed, and clinical progression. Tau burden strongly relates to baseline episodic and semantic memory, which may reflect that it is heavily weighted by entorhinal tau, a region previously linked to memory processing. In contrast, stronger associations between tau spread extent and baseline attention/processing speed could reflect the inclusion of additional brain regions, particularly the frontal lobe, which support a wider range of cognitive processing. Additionally, tau spread extent is generally more sensitive to baseline preclinical deficits; however, tau burden better estimates future decline across all cognitive domains and clinical symptom onset. Together, these findings suggest complementary utility of evaluating both tau burden and tau spread extent in early AD progression.

## 1 Introduction

Understanding how Alzheimer disease (AD) progresses across its disease course is crucial for early detection and for successful intervention and treatment (Brookmeyer et al., 2018;Congdon & Sigurdsson, 2018;Jack et al., 2018;Nelson et al., 2012). AD is a neurodegenerative disease characterized by the development of amyloid-beta (Aβ) plaques and tau-protein neurofibrillary tangles (NFTs) (Gordon et al., 2018;Zhang et al., 2021). While Aβ plaques develop at early stages of AD (Nordberg, 2004), cognitive impairment and neurodegeneration are observed as tau NFTs begin to develop (Brier et al., 2016;Nelson et al., 2012) with a strong spatial correlation (Gordon et al., 2018;La Joie et al., 2020). Previous histopathological studies demonstrated a progressive spatial pattern of tau in AD (Braak & Braak, 1991) beginning in the entorhinal cortex, expanding to the medial temporal lobe, and finally reaching additional neocortical regions (Gordon et al., 2016;Takeda, 2019). This pattern of spread aligns with typical amnestic AD cognitive impairment, beginning with memory deficits and later widespread dysfunction. The spatiotemporal pattern of tau progression can be deconstructed into two key components: initial tau accumulation in early stage regions (tau burden) and spread into new regions (tau spread).

More recently, tau has been studied*in vivo*using positron emission tomography (PET). Tau PET researchers have typically quantified tau tracer uptake with standard uptake value ratios (SUVRs) in three ways: a summary measure focused on regions that accumulate tau early in the disease (Cho et al., 2016;Jack et al., 2017;Mishra et al., 2017), by evaluating multiple regions of interest (ROIs) that align with Braak staging (Braak & Braak, 1991), or by using regions selected to standardize quantification across different tau PET tracers (Villemagne et al., 2023). Such methods have been developed to be sensitive to early stages of clinical impairment but are tuned to typical amnestic presentations of AD and corresponding tau spatiotemporal pathological progression. However, domain-specific cognitive deficits related to spatial patterns of tau have been identified in many individuals (Bejanin et al., 2017;Ossenkoppele et al., 2016) that are distinct from the typical amnestic signatures.

Subgroups with AD pathology but distinct nonamnestic cognitive profiles have been demonstrated throughout AD literature (Alladi et al., 2007;Duara & Barker, 2022;Lam et al., 2013). These variants have often been classified as atypical patterns of AD or have been associated with other pathological diseases such as frontotemporal degeneration (FTD) or Lewy body disease (Crutch et al., 2012;Johnson et al., 1999;Meyer et al., 2015;Ossenkoppele et al., 2016;Preiß et al., 2019;Sawyer et al., 2017). Three primary variants have been identified based on the predominant localization of tau pathology: (1) a visuospatial variant with posterior cortical atrophy (PCA), (2) a language variant with logopenic variant primary progressive aphasia (lvPPA), and (3) a behavioral variant that impacts frontal regions (bvAD)

Recent studies have progressively demonstrated the heterogeneity of pathological and clinical presentations of AD (Jellinger, 2022). Real world populations present with variable phenotypes regardless of AD subtype classification. Further, individual patients present much more complex biological relationships than are captured in group-averaged spatial patterns of pathology. AD is more prevalent in older adults, who demonstrate high frequency of comorbidities that may affect AD pathology in ways that are not well understood. Robust evaluation accounting for spatial variability of tau deposition is, therefore, important for patient-specific assessment.

Given strong support for heterogeneity of AD tau accumulation, SUVR summary measures focusing on tau burden in “early” Braak regions, identified based on group-averaged amnestic AD and autopsy studies, may, therefore, be insufficient for evaluating AD progression and subsequent cognitive impairment beyond memory. These methods may not adequately account for variability between subgroups and individuals, and may contribute to participant misclassification or misdiagnosis. To address this limitation, our previous work (Doering et al., 2024) quantified the extent of tau spread throughout the brain, or Tau Spatial Spread (TSS). TSS is correlated with a conventional summary measure of tau burden, Tau Index (TI), calculated based on traditional “early” regions for amnestic AD, but has greater variability than TI in preclinical AD. As TSS equally weighs all regions in the brain, it may capture tau pathology for atypical AD variants better than TI and may be robust to interindividual tau spatial heterogeneity.

In this work, we evaluate how TI and TSS relate to general cognition using the Knight Preclinical Alzheimer Cognitive Composite (Knight PACC), as well as specific cognitive domains, using composite scores of episodic memory, semantic memory, working memory, and attention/processing speed for individuals at early stages of AD. We hypothesize that memory composites are more closely related to early tau burden (TI) since episodic memory is linked to regions that initially develop AD tau pathology. We also hypothesize that tau spread (TSS) will account for tau pathology in more disparate regions even at early stages of AD, capturing more broadly distributed network dysfunction and decreased performance in the attention/processing speed composite.

## 2 Methods

### 2.1 Participants

Data used in this study were collected from participants enrolled in ongoing studies of memory and aging from the Charles F. and Joanne Knight Alzheimer Disease Research Center (Knight ADRC) at Washington University School of Medicine (WUSM) between 2014 and 2023 in the 22^nd^data freeze (DF22). Demographic information such as age, sex, race, and education were self-reported by participants.

#### 2.1.1 Older adults

To be considered eligible for the overall study, older adults were required to be at least 50 years old with both tau PET and structural MRI (n = 535). Additional eligibility criteria were imposed for each analysis based on specific data availability, which allowed us to maximize sample size and thus statistical power. For inclusion in cross-sectional cognitive analyses, individuals were required to have been evaluated using the Clinical Dementia Rating® (CDR®) (Hughes et al., 1982;J. C. Morris, 1993), completed an amyloid PET scan, and completed the standard Knight ADRC neuropsychological battery within 12 months of their tau PET scan (n = 489). For inclusion in longitudinal cognitive analyses, individuals were once again required to have completed the neuropsychological battery within 12 months of their tau PET scan but must additionally have completed the neuropsychological battery in at least one follow-up visit (n = 444). Finally, individuals who were assessed to be cognitively unimpaired (CDR = 0) during their baseline clinical assessment and who had completed follow-up clinical visits were utilized for analyses focused on how these variables interact with longitudinal clinical status (n = 403).

#### 2.1.2 Younger adults

In order to robustly estimate measures of tau spread extent, younger adults identified from both the Knight ADRC cohort (n = 9) and the Dominantly Inherited Alzheimer Network (DIAN)’s Observational study (DF16; n = 29) (McKay et al., 2023) were included as young controls (YC). For both cohorts, individuals were required to be aged 49 years or younger, be assessed as cognitively unimpaired (CDR = 0), and have been determined to be amyloid negative using a validated amyloid PET cutoff (Su et al., 2013,^2019^). Moreover, individuals recruited from DIAN needed to be noncarriers of autosomal dominant AD mutations (*PSEN1*,*PSEN2*,*APP*) (Moulder et al., 2013).

### 2.2 Ethics

All participants provided written informed consent and the process for data collection was approved by the Washington University Human Research Protection Office, which serves as the central institutional review board (IRB), for the Knight ADRC and DIAN studies.

### 2.3 Imaging acquisition and processing

#### 2.3.1 Structural MRI

T1-weighted MRI scans were acquired on a DIAN-approved 3T scanner at a resolution of either 1 x 1 x 1.25 mm^3^or 1 x 1 x 1 mm^3^. Cortical and subcortical ROIs for PET analyses were defined from the structural T1 using FreeSurfer (v5.3-HCP;http://surfer.nmr.mgh.harvard.edu/) (Fischl, 2012).

#### 2.3.2 Amyloid PET

Amyloid PET imaging was performed using 10.02 ± 0.60 mCi of^18^F-florbetapir (AV-45) or 15.13 ± 3.76 mCi of^11^C-Pittsburgh Compound B (PiB). For AV-45, regional standard uptake value ratios (SUVRs) were calculated using the cerebellar gray as the reference region for the 50–70 minute postinjection window using the FreeSurfer-based PET Unified Pipeline (PUP;https://github.com/ysu001/PUP) (Su et al., 2013). For PiB, the postinjection window for SUVR quantification was dependent on the cohort source (30–60 minutes for Knight ADRC and 40–70 minutes for DIAN).

Global amyloid burden was evaluated with a cortical summary measure by averaging partial volume-corrected SUVR across precuneus, prefrontal cortex, gyrus rectus, and lateral temporal cortex ROIs. Amyloid positivity was determined using our previously published thresholds for AV-45 (SUVR > 1.19) and PiB (SUVR > 1.42) (Su et al., 2013,^2019^).

#### 2.3.3 Tau PET

Tau PET imaging was performed using 9.06 ± 0.87 mCi of^18^F-flortaucipir (AV-1451). Regional and voxel-specific SUVRs were calculated for the 80–100 minute postinjection window using PUP. A partial volume correction technique is implemented in the processing pipeline for regional SUVRs using a regional spread function (RSF) (Rousset et al., 1998)-based approach (Su et al., 2015). Rigorous quality control is conducted at several stages including visual inspection of raw scans for motion and artifacts, manual correction of FreeSurfer segmentation, and visual inspection of postprocessed images for abnormalities and alignment.

**Fig. 1.**
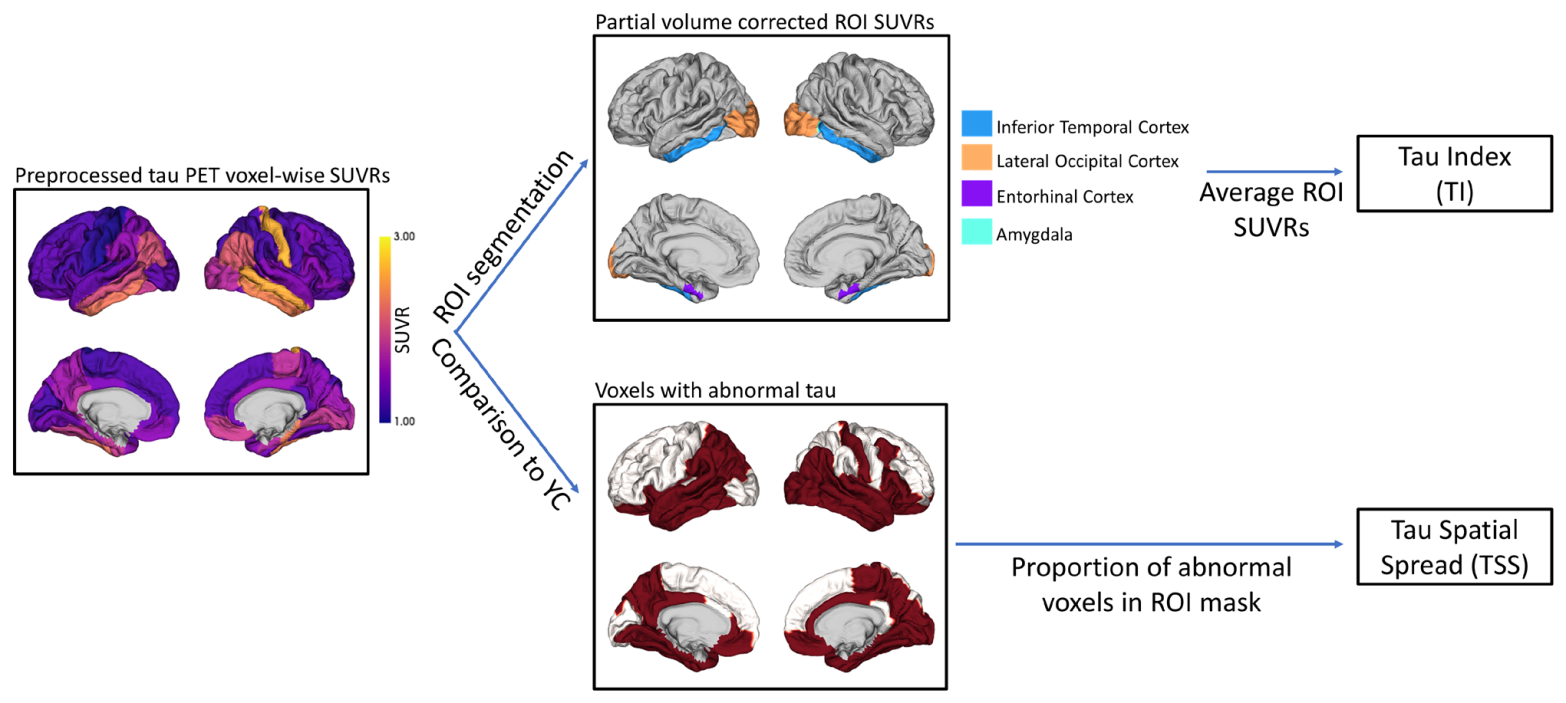
Schematic for the quantification of Tau Index and Tau Spatial Spread. Participant tau PET scans are first preprocessed to calculate voxel-wise SUVR images. These images are then processed in two independent pipelines to calculate Tau Index (TI) and Tau Spatial Spread (TSS). First, participant structural MRIs are segmented in FreeSurfer and applied to the participant SUVR image to calculate partial volume-corrected SUVRs for four regions of interest: the inferior temporal cortex, lateral occipital cortex, entorhinal cortex, and amygdala. TI is then calculated as the mean of the resulting regional SUVRs. Second, participant structural MRIs and SUVR images are compared with younger control scans in order to identify voxels with abnormal tau pathology in a process previously published (Doering et al., 2024). TSS is then calculated as the proportion of abnormal voxels within biologically relevant brain regions.

Two tau metrics were calculated in parallel processing pipelines (Fig. 1). Tau index (TI) was calculated as the mean regional SUVR for four regions previously identified (Mishra et al., 2017) to characterize early tau accumulation (entorhinal cortex, amygdala, lateral occipital cortex, and inferior temporal cortex). Tau spatial spread (TSS) was measured from voxel-wise tau PET images as the proportion of voxels within the cortex, hippocampus, and amygdala with abnormal tau pathology (z > 1.96) relative to YC tau PET scans. The method for processing the tau PET scans to calculate TSS is described in more detail in our previously published work (Doering et al., 2024).

### 2.4 Neuropsychological testing

Since 2005, neuropsychological evaluation at the Knight ADRC has primarily utilized Uniform Data Set (UDS) tests (Beekly et al., 2007;J. C. Morris et al., 2006;Weintraub et al., 2009). For our analyses, this large cognitive battery was reduced to five cognitive composite values: (1) Knight PACC, (2) episodic memory, (3) semantic memory, (4) working memory, and (5) attention/processing speed. The creation of these composites is outlined in a recent paper (McKay et al., 2024). Using an equipercentile equating method previously validated (Monsell et al., 2016), tasks from UDS versions 2 and 3 were equated to produce compatible scores across a changing test battery. The Delayed Craft Story task (Craft et al., 1996) was equated with the Delayed Logical Memory IIA task (Wechsler, 1997a,1997b), the Multilingual Naming Test (Ivanova et al., 2013) was equated with the Boston Naming Test (Goodglass & Kaplan, 1983), and the Number Span Forward/Backward tasks (Weintraub et al., 2009) were equated with the Digit Span Forward/Backward tasks (Wechsler, 1997a,1997b).

The Knight PACC composite is derived from the free recall score of the Free and Cued Selective Reminding Test (Grober et al., 1998), the total correct score from the Digit Symbol subtest of the WAIS-R (Wechsler, 1981), the total completion time from the Trail Making Test Part B (Armitage, 1946), and the total correct score from the Animal Naming Test (Goodglass & Kaplan, 1983). The episodic memory composite is derived from the total score of the Delayed Logical Memory IIA task (Wechsler, 1997a,1997b), the free recall score of the Free and Cued Selective Reminding Test (Grober et al., 1998), and the total score of the WMS-R’s Associate Memory tasks (Wechsler, 1997a,1997b). The semantic memory composite is derived from the total scores for the Vegetable Naming Test, the Animal Naming Test, and the Boston Naming Test (Goodglass & Kaplan, 1983). The working memory composite was derived from the total scores of the Digit Span Forward and Backward tasks, as well as the Letter-Number Sequencing task (Wechsler, 1997a,1997b). The attention/processing speed composite is derived from the time to complete Trail Making Test parts A and B (Armitage, 1946), the total score of the Digit Symbol subtask from the WAIS-R (Wechsler, 1981), and the total score Consonant-Vowel Odd-Even (CVOE) switching task (Huff et al., 2015).

After standardizing each participant’s raw score on each task to the mean and standard deviation of the scores recorded for the cognitively normal older adults’ baseline visit, unweighted z-scores for each group of tasks were averaged together to form each composite score. Composite scores were calculated for all available visits in all participants regardless of cognitive status and were entered as the cognitive variables of interest for all subsequent analyses. Standardized scores for the subtasks of the Trail Making Test were reverse scored to ensure that in all cases higher composite scores represented better task performance.

### 2.5 Clinical testing

Clinical evaluation was conducted with the CDR®, a tool for assessing the presence and, when present, the severity of dementia. The CDR evaluates whether there has been a change from previously attained levels of function in six domains—memory, orientation, judgment and problem solving, community affairs, home and hobbies, and personal care. The global CDR is scored on a 5-point scale that denotes no impairment (CDR = 0), very mild impairment (CDR = 0.5), mild impairment (CDR = 1), moderate impairment (CDR = 2), and severe impairment (CDR = 3).

### 2.6 Disease-stage classification

Participants were assigned to disease stage dependent on amyloid positivity (Aβ+) and clinical status (CDR), resulting in three groups for analysis: Aβ-CDR0 (older control, OC), Aβ+CDR0 (preclinical AD), and Aβ+CDR>0 (symptomatic AD). Participants identified as Aβ-CDR>0 were excluded from analyses. Disease-stage groups correspond to early clinical progression of AD, spanning the continuum of amyloid-beta plaque accumulation and cognitive decline.

### 2.7 Statistical analyses

Analyses were conducted with R (v4.1.0). Significance testing was conducted with threshold p < 0.05. Multiple comparisons correction was applied to all pairwise or group comparisons with Benjamini–Hochberg (BH) procedure with a false discovery rate of 0.05.

The relative estimative strength of TI and TSS for the following analyses is evaluated with the seminested framework of four models: (1) covariate model, (2) TI model, (3) TSS model, and (4) additive model (in which both TI and TSS are included as variables). All models include covariates of age, sex, and education. Model comparisons are conducted by calculating corrected AIC (AICc) to identify the most plausible model, penalized for extra variables. The AICc weight represents the relative likelihood of each model. The model with the lowest AICc indicates the “best” model, however, a difference in AICc of <2 demonstrates that both models are comparable (Burnham & Anderson, 2004). ΔAICc is calculated for each model relative to the best model as ΔAICc = AICc – min(AICc).

#### 2.7.1 Baseline tau and baseline cognition

Cognitive composite scores, TI, and TSS were evaluated between disease-stage groups using Wilcoxon–Mann–Whitney tests. BH correction was applied for pairwise comparisons between disease stages.

Cognitive composite scores were then evaluated relative to TI and TSS separately for each disease-stage group with Spearman correlations. BH correction was applied for comparison of correlations between disease stages.

The relative strength of TI and TSS to estimate each cognitive composite at baseline was evaluated with the seminested model framework. For each composite (comp), the subject data were fit by linear regression with baseline covariates age, sex, and education (educ), resulting in the following models:

Eq. 1.1: Covariate Model

comp~age+sex+educ+ε.

Eq. 1.2: TI Model

comp~TI+age+sex+educ+ε.

Eq. 1.3: TSS Model

comp~TSS+age+sex+educ+ε.

Eq. 1.4: Additive Model

comp~TI+TSS+age+sex+educ+ε.

#### 2.7.2 Baseline tau and longitudinal cognition

The relative strength of TI and TSS to estimate longitudinal change for each cognitive composite was evaluated with the seminested model framework. For each composite (comp), the subject (sub) data for all time points were fit by linear mixed effects regression with time as a random effect and baseline covariates age, sex, and education (educ) as fixed effects, resulting in the following models:

Eq. 2.1: Covariate Model

$$\begin{array}{l} \text{comp}\sim (\text{time}|\text{sub})+\text{age}\;\text{*}\;\text{time}+\text{sex}\;\text{*}\;\text{time}+\text{educ}\;\text{*}\;\\ \;\;\;\;\text{time}+\varepsilon . \end{array}$$

Eq. 2.2: TI Model

$$\begin{array}{l} \text{comp}\sim (\text{time}|\text{sub})+\text{TI}\;\text{*}\;\text{time}+\text{age}\;\text{*}\;\text{time}\\ \;\;\;+\text{sex}\;\text{*}\;\text{time}+\text{educ}\;\text{*}\;\text{time}+\varepsilon . \end{array}$$

Eq. 2.3: TSS Model

$$\begin{array}{l} \text{comp}\sim (\text{time}|\text{sub})+\text{TSS}\;\text{*}\;\text{time}+\text{age}\;\text{*}\;\text{time}\\ \;\;\;+\text{sex}\;\text{*}\;\text{time}+\text{educ}\;\text{*}\;\text{time}+\varepsilon . \end{array}$$

Eq. 2.4: Additive Model

$$\begin{array}{l} \text{comp}\sim (\text{time}|\text{sub})+\text{TI}\;\text{*}\;\text{time}+\text{TSS}\;\text{*}\;\text{time}+\text{age}\;\text{*}\;\text{time}\\ \;\;\;+\text{sex}\;\text{*}\;\text{time}+\text{educ}\;\text{*}\;\text{time}+\varepsilon . \end{array}$$

Estimated Marginal Means (EMM) were calculated for the TI model and TSS model to demonstrate the estimated rate of change in the composites based on each tau metric at baseline. Gaussian Mixture Modeling (GMM) was conducted assuming two underlying distributions for each of baseline TI and baseline TSS, independently. Subsequently, the means of the two resulting distributions within each measure (Supplemental Fig. 1) were implemented into the EMM models as representative “low” and “high” values for TI and TSS.

#### 2.7.3 Baseline tau and longitudinal clinical evaluation

Clinical progression was evaluated using survival models for individuals with baseline CDR=0, in which an event is the first occurrence where CDR progressed above 0. Kaplan–Meier curves were developed by splitting participants into two groups dependent on either baseline TI or baseline TSS, split by the median value for the modeled individuals.

The relative strength of TI and TSS to estimate longitudinal clinical survival was evaluated with the seminested model framework. Subject data were fit by Cox proportional hazards regression predicting survival (CDR = 0) status at time (t) with baseline covariates age, sex, and education (educ), resulting in the following models:

Eq. 3.1: Covariate Model

survival(time, status)~age+sex+educ.

Eq. 3.2: TI Model

survival(time, status)~TI+age+sex+educ.

Eq. 3.3: TSS Model

survival(time, status)~TSS+age+sex+educ.

Eq. 3.4: Additive Model

survival(time, status)~TI+TSS+age+sex+educ.

## 3 Results

In total, 38 YC and 535 older participants were identified with baseline tau PET and MRI which passed quality control. In total, 479 of the older participants were additionally classified into a disease-stage group (OC, Preclinical AD, Symptomatic AD) based on amyloid PET and CDR. Summary descriptives for these disease-stage groups and YC are provided inTable 1.

**Table 1.** Participant demographics.

	Younger control	Older control	Preclinical AD	Symptomatic AD
N	38	287	137	55
Age, years	37.89 (9.04)	68.80 (8.24)	71.31 (7.24)	75.09 (6.52)
Sex				
Female	27 (71%)	146 (51%)	93 (68%)	27 (49%)
Male	11 (29%)	141 (49%)	44 (32%)	28 (51%)
MMSE	29.10 (0.92)	29.29 (1.07)	29.21 (1.18)	25.28 (3.74)
CDR				
0	38 (100%)	287 (100%)	137 (100%)	
0.5				44 (80%)
1				11 (20%)
Education, years	16.16 (2.19)	16.53 (2.29)	16.49 (2.12)	15.24 (2.73)
Race				
AIAN			1 (<1%)	
Asian		2 (1%)	1 (<1%)	
Black	2 (5%)	39 (14%)	11 (8%)	3 (5%)
White	19 (50%)	245 (85%)	124 (91%)	51 (93%)
More than 1				1 (2%)
Unknown	17 (45%)			
APOE ε4				
Carrier	12 (32%)	90 (31%)	61 (45%)	37 (67%)
Noncarrier	26 (68%)	196 (68%)	76 (55%)	18 (33%)
Unknown		1 (<1%)		

Participant sample size and characteristics for healthy controls and participants, split by disease-stage groups. Data are formatted as n (%) or mean (SD).

### 3.1 Baseline tau and baseline cognition

In total, 477 older participants were identified with tau PET, neuropsychological testing, and classified into disease-stage groups. Cognitive composite scores were calculated relative to the OC group. Participants with insufficient neuropsychological testing for a cognitive composite were excluded from corresponding analyses.

**Fig. 2.**
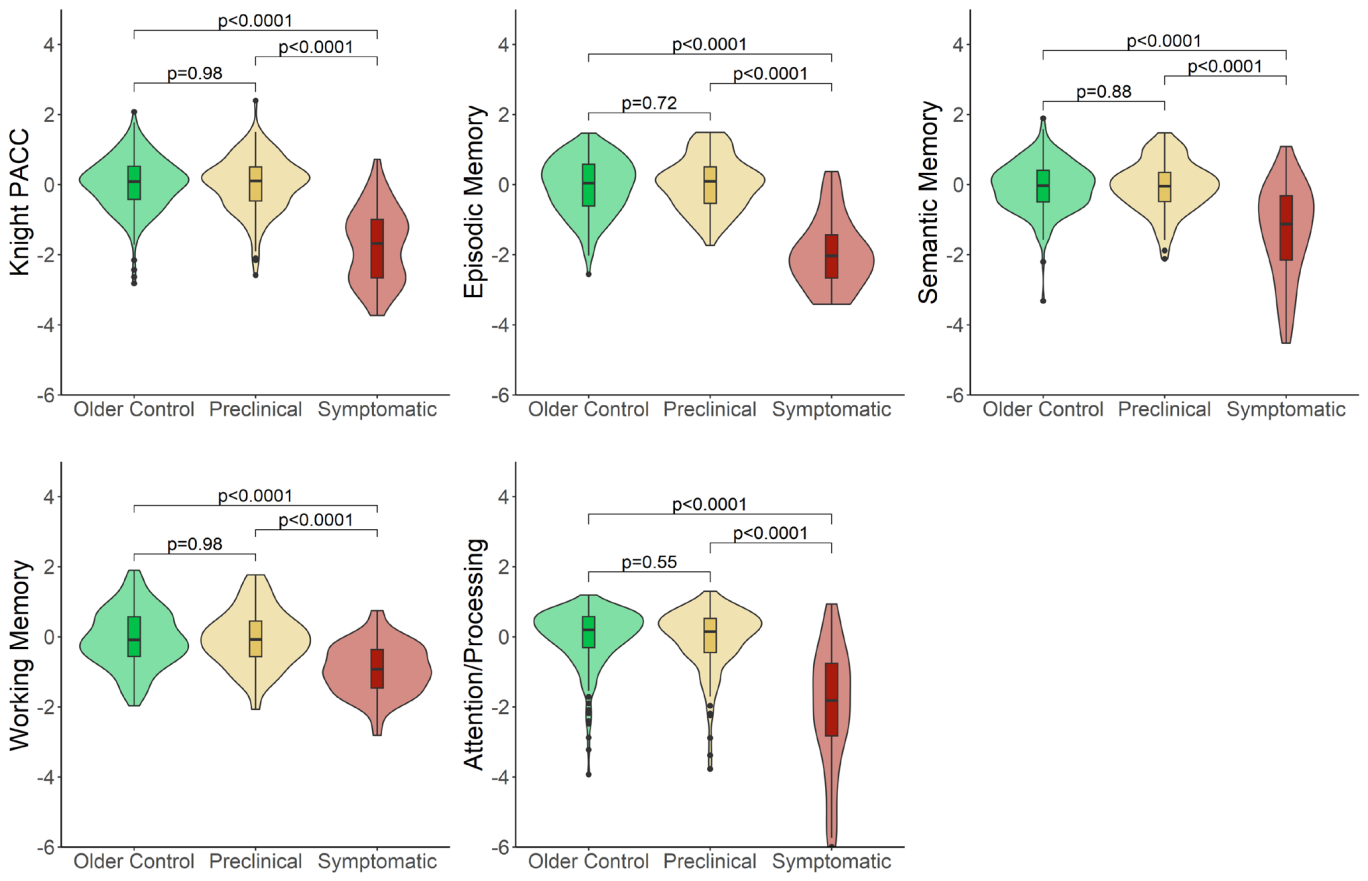
Baseline cognitive performance per disease-stage groups. Comparison of baseline cognitive domain composite scores across disease-stage groups. Pairwise comparisons conducted using Wilcoxon–Mann–Whitney U tests with multiple comparison correction using Benjamini–Hochberg procedure.

Cognitive composite scores were evaluated between disease-stage groups (Fig. 2). For all composites, the Symptomatic group performed worse than the OC and Preclinical groups (p < 0.0001). No significant difference was observed between the OC and Preclinical groups (Supplemental Table 1).

**Fig. 3.**
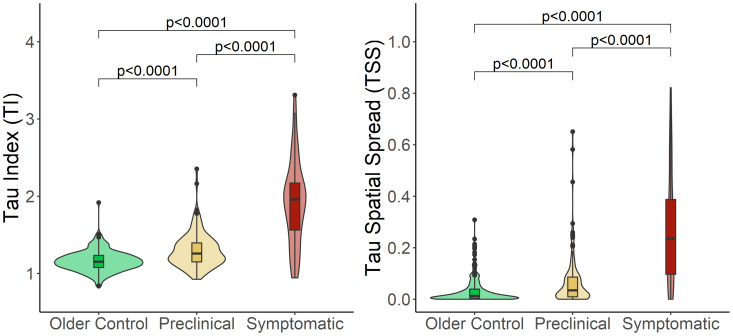
Tau Index and Tau Spatial Spread per disease-stage groups. Comparison of Tau Index and Tau Spatial Spread across disease-stage groups. Pairwise comparisons conducted using Wilcoxon–Mann–Whitney U tests with multiple comparison correction using Benjamini–Hochberg procedure.

TI and TSS were evaluated between disease-stage groups (Fig. 3). The Symptomatic group showed higher levels of TI and TSS than the OC and Preclinical groups (p < 0.0001). The Preclinical group additionally showed higher levels of TI and TSS than the OC group (p < 0.0001) (Supplemental Table 2). Distribution summaries of TI and TSS are provided inSupplemental Figure 2for all disease-stage groups and YC.

**Fig. 4.**
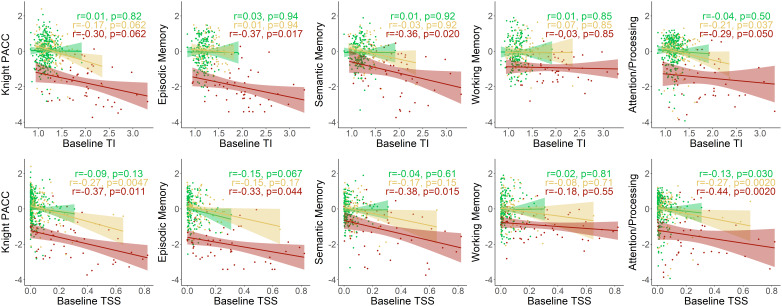
Baseline cognitive performance relative to Tau Index or Tau Spatial Spread per disease-stage group. Comparison of baseline cognitive domain composite score and TI (top) or TSS (bottom) per disease-stage group (green: OC; yellow: Preclinical AD; red: Symptomatic AD). Spearman correlations reported with multiple comparison correction using Benjamini–Hochberg procedure per disease-stage group. Shaded area refers to 95% confidence interval of the regression slope.

Cognitive composite scores were then evaluated against TI and TSS for each disease-stage group (Fig. 4;Supplemental Table 3). For the OC group, there was no association between any composite and TI. There was an association between the attention/processing speed composite and TSS (R = -0.13, p = 0.030). For the Preclinical group, there was an association between TI and the attention/processing speed composite (R = -0.21, p = 0.037) as well as associations between TSS and the Knight PACC (R = -0.27, p = 0.0047) and attention/processing speed composites (R = -0.27, p = 0.0020). A trend was observed between TI and the Knight PACC but was not significant after multiple comparisons correction. For the Symptomatic group, there was an association for both TI and TSS with the episodic memory composite (TI: R = -0.37, p = 0.017; TSS: R = -0.33, p = 0.044) and the semantic memory composite (TI: R = -0.36, p = 0.020; TSS: R = -0.38, p = 0.015). TSS was additionally associated with the Knight PACC (R = -0.37, p = 0.011) and the attention/processing speed composite (R = -0.44, p = 0.0020). Trends were observed between TI and both the Knight PACC and the attention/processing speed composite but were not significant after multiple comparisons correction.

Model comparisons were conducted for estimating baseline score of the five cognitive composites using the seminested model framework (Table 2;Supplemental Table 4). For all composites, the covariate models, as assessed by AICc, performed worse than the TI and TSS models, indicating there is added benefit of modeling tau when estimating baseline cognitive performance. The additive models performed best for the Knight PACC, episodic memory, semantic memory, and attention/processing speed composites. When comparing the TI and TSS models directly, we find that TSS outperforms TI for the Knight PACC (ΔAICc = 4.34) and attention/processing speed composite (ΔAICc = 17.43), but TI outperforms TSS for the episodic memory (ΔAICc = 10.76) and semantic memory (ΔAICc = 4.81) composites. These results indicate that TI is better associated with memory-specific cognitive deficits, while TSS is better associated with executive deficits. However, the inclusion of both metrics provides the strongest estimative power.

**Table 2.** Baseline cognitive model comparison.

	Model	K	AICc	Δ AICc	AICc Weight	Log-Likelihood
PACC	Additive	7	1071.26	0.00 ***	0.98	-528.51
	TSS	6	1079.05	7.79	0.02	-533.44
	TI	6	1083.39	12.13	0.00	-535.60
	Covariate	5	1193.58	122.31	0.00	-591.73
Episodic	Additive	7	855.33	0.00 ***	0.87	-420.49
	TI	6	859.20	3.88	0.13	-423.47
	TSS	6	869.96	14.63	0.00	-428.85
	Covariate	5	965.65	110.32	0.00	-477.73
Semantic	Additive	7	789.31	0.00 ***	0.84	-387.48
	TI	6	792.87	3.56	0.14	-390.31
	TSS	6	797.68	8.38	0.01	-392.71
	Covariate	5	872.52	83.22	0.00	-431.17
Working	TSS	6	799.26	0.00 ***	0.71	-393.50
	Additive	7	801.33	2.06	0.25	-393.49
	TI	6	805.49	6.23	0.03	-396.62
	Covariate	5	820.07	20.81	0.00	-404.94
Attention	Additive	7	1248.15	0.00 ***	0.84	-616.95
	TSS	6	1251.48	3.33	0.16	-619.65
	TI	6	1268.91	20.75	0.00	-628.36
	Covariate	5	1376.98	128.82	0.00	-683.42

Comparison of covariate, individual TI, individual TSS, and additive models estimating baseline cognitive domain composite scores. Linear regressions with covariates age, sex, and education evaluated with log-likelihood and corrected AIC (AICc). Model nominated by AICc (***) and comparable models (*) identified for each cognitive domain composite. Models ordered by AICc with the nominated model listed first.

### 3.2 Baseline tau and longitudinal cognition

In total, 444 older participants were identified with tau PET and neuropsychological testing, as well as follow-up neuropsychological testing after the baseline visit (Table 3). Participants with insufficient neuropsychological testing for a cognitive composite were excluded from corresponding analyses.

**Table 3.** Participant follow-up cognitive and clinical visits.

	PACC	Episodic	Semantic	Working	Attention	CDR
N	371	373	381	380	367	403
Observations	1239	1560	1595	1592	1228	1969
Visits per subject						
2	118	75	77	77	115	61
3	110	80	79	78	109	42
4	76	70	74	74	77	63
5	40	60	61	61	40	84
6	20	51	52	52	19	71
7	7	14	15	15	7	44
8		21	21	21		27
9		2	2	2		11
Follow-up visit time since baseline, years	2.69 (1.57)	3.27 (1.70)	3.26 (1.69)	3.26 (1.69)	2.69 (1.56)	3.48 (1.76)
Time between visits, years	1.52 (0.85)	1.14 (0.44)	1.14 (0.42)	1.13 (0.42)	1.52 (0.85)	1.24 (0.62)

Summary of longitudinal participant time points available for cognitive domain composites and CDR. Data are formatted as N observations or mean (SD).

Model comparisons were conducted for estimating longitudinal score of the five cognitive composites using the seminested model framework (Table 4;Supplemental Table 5). For all composites, the covariate models, as assessed by AICc, performed worse than the TI and TSS models, indicating there is added benefit of modeling tau when estimating future cognitive performance. The additive models performed best for the episodic memory and attention/processing speed composites. The TI model performs better than the TSS model across all composites. These results indicate that TI estimates longitudinal cognitive performance better than TSS. EMMs for the TI and TSS models are depicted inFigure 5.

**Table 4.** Longitudinal cognitive model comparison.

	Model	K	AICc	Δ AICc	AICc Weight	Restricted Log-Likelihood
PACC	TI	14	1897.83	0.00 ***	0.54	−934.74
	Additive	16	1898.16	0.33 *	0.46	−932.86
	TSS	14	1922.61	24.78	0.00	−947.13
	Covariate	12	2015.38	117.55	0.00	−995.56
Episodic	Additive	16	2763.17	0.00 ***	0.86	−1365.41
	TI	14	2766.72	3.55	0.14	−1369.23
	TSS	14	2862.71	99.54	0.00	−1417.22
	Covariate	12	2949.37	186.20	0.00	−1462.58
Semantic	TI	14	2694.55	0.00 ***	0.74	−1333.14
	Additive	16	2696.66	2.11	0.26	−1332.16
	TSS	14	2758.44	63.89	0.00	−1365.09
	Covariate	12	2899.13	204.58	0.00	−1437.47
Working	TI	14	2588.63	0.00 ***	0.86	−1280.18
	Additive	16	2592.40	3.77	0.13	−1280.03
	TSS	14	2597.95	9.32	0.01	−1284.84
	Covariate	12	2621.87	33.24	0.00	−1298.84
Attention	Additive	16	2412.14	0.00 ***	0.63	−1189.84
	TI	14	2413.40	1.26 *	0.33	−1192.53
	TSS	14	2417.77	5.63	0.04	−1194.71
	Covariate	12	2487.99	75.85	0.00	−1231.87

Comparison of covariate, individual TI, individual TSS, and additive models estimating longitudinal visit cognitive domain composite scores. Linear mixed-effects regressions with covariates age, sex, and education evaluated with restricted log-likelihood and corrected AIC (AICc). Model nominated by AICc (***) and comparable models (*) identified for each cognitive domain composite. Models ordered by AICc with the nominated model listed first.

**Fig. 5.**
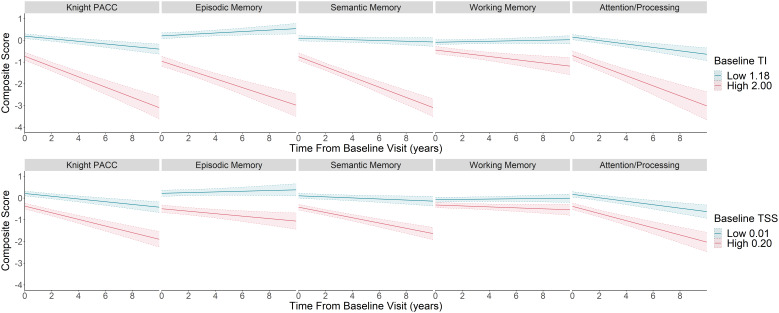
Longitudinal cognitive performance estimated from baseline Tau Index and Tau Spatial Spread. Estimated longitudinal cognitive domain composite score performance dependent on baseline TI (top) or TSS (bottom). Estimated Marginal Means calculated from linear mixed effect regressions (TI Model and TSS Model) accounting for covariates age, sex, and education. Representative “low” and “high” values identified for TI and TSS from the participant sample.

### 3.3 Baseline tau and longitudinal clinical evaluation

In total, 403 older participants were identified with tau PET and clinical testing (CDR = 0 at baseline), as well as follow-up clinical testing after the baseline visit (Table 3). Survival analyses indicate 60 participants experience “events” where follow-up CDR progressed above 0. The remaining 343 participants are right censored since they remain CDR0 throughout all follow-up visits. CDR survival is depicted inFigure 6(Supplemental Table 6) using a median split (TI = 1.18, TSS = 0.019).

**Fig. 6.**
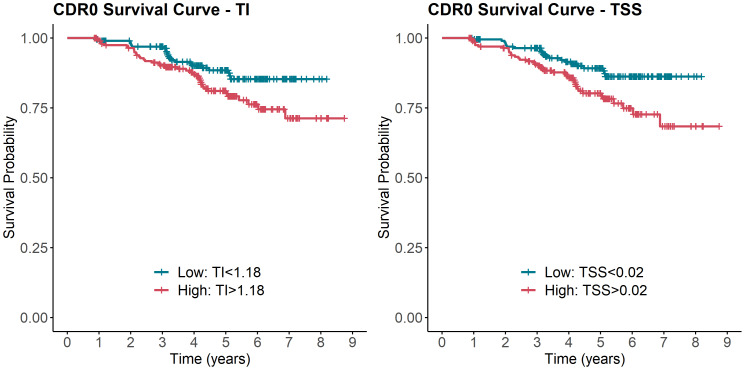
Longitudinal clinical progression evaluated by baseline Tau Index and Tau Spatial Spread. Clinical progression evaluated for cognitively normal individuals (CDR = 0) at baseline with Kaplan–Meier survival curves where an event is classified as the first occurrence where participants demonstrate AD symptoms (CDR > 0). Survival is compared for participants with low/high TI (left) or TSS (right), categorized by median split of the participant sample.

Model comparisons were conducted for estimating longitudinal CDR survival using the seminested model framework (Table 5;Supplemental Table 7). The TI model performed best, as assessed by AICc, but there is substantial support for the additive model (ΔAICc = 1.20). The TSS model (ΔAICc = 17.56) demonstrates minor improvement over the covariate model (ΔAICc = 22.46), indicating that TSS is minimally predictive of future clinical conversion and that TI is a much stronger metric for estimating future clinical progression.

**Table 5.** Longitudinal clinical model comparison.

	Model	K	AICc	Δ AICc	AICc weight	Log-likelihood
CDR	TI	4	624.61	0.00 ***	0.65	−308.25
	Additive	5	625.81	1.20 *	0.35	−307.83
	TSS	4	642.17	17.56	0.00	−317.03
	Covariate	3	647.07	22.46	0.00	−320.50

Comparison of covariate, individual TI, individual TSS, and additive models estimating longitudinal CDR score. Cox proportional hazards regressions with covariates age, sex, and education evaluated with log-likelihood and corrected AIC (AICc). Model nominated by AICc (***) and comparable models (*) identified.

## 4 Discussion

In this study, we aimed to evaluate the relationship between tau pathology, independently quantified as burden and spread, and domain-specific cognitive performance for individuals in the early stages of AD. Our results demonstrate elevated baseline TI and TSS across all disease stages, including between older controls and preclinical AD participants. However, decreased performance in cognitive domain composites between groups is only observed once individuals become symptomatic. This is consistent with several studies demonstrating the onset of tau pathological accumulation after widespread amyloid plaque accumulation but prior to clinical diagnosis observed in preclinical AD (Bloom, 2014;Gulisano et al., 2018). Tau pathology is tightly coupled to neurodegeneration and cognitive impairment in Alzheimer disease (Ossenkoppele et al., 2019), and studies show that advancing tau pathology degrades higher level cognitive functions in patients with AD (Bocancea et al., 2023). However, the differentiation between tau burden and tau spread in relation to domain-specific cognitive deficits is not yet well understood. To address this gap, we investigated whether elevated tau burden and tau spread are negatively correlated with cognitive performance in general cognition (Knight PACC) as well as four cognitive domain composites: episodic memory, semantic memory, working memory, and attention/processing speed.

Deficits in episodic memory, semantic memory, and attention/processing speed have all been previously identified in AD patients (Gallagher & Koh, 2011;Hennawy et al., 2019;Rogers & Friedman, 2008). Episodic memory has long been the focus for identifying early cognitive decline in AD; however, deficits have been observed across several cognitive domains as early as preclinical AD (Bäckman et al., 2005;Gollan et al., 2024;Small et al., 2003). In this study, both TI and TSS were negatively correlated with cognitive domain composite scores for the symptomatic AD participants. However, TI was more sensitive to deficits in episodic and semantic memory, while TSS was more sensitive to deficits in Knight PACC and attention/processing speed. We additionally found that TSS was better able to capture deficits in attention/processing speed before the onset of clinical symptoms, as observed in preclinical AD participants.

Our findings suggest differential utility of TI and TSS in evaluating early AD cognitive impairment. This difference in utility corresponds to the conceptual difference between TI, a standard SUVR metric, and TSS, the proposed metric, and the neural correlates of cognition captured by each tau metric. That is, TI is a measure of tau burden, focusing on regions that develop tau pathology early in the disease for an amnestic AD cohort, while TSS is a measure of tau spread, including all cortical regions without regard to how much tau pathology continues to develop in impacted regions. TSS, therefore, can capture deficits related to neural correlates outside of the scope of TI; however, it cannot account for the ongoing tau tangle development in early regions that may further affect cognitive ability.

TI is particularly sensitive to deficits in the episodic memory and semantic memory composites. Leading models of episodic memory identify important roles of the medial temporal lobe (Nadel & Moscovitch, 1997;Squire & Alvarez, 1995). The entorhinal cortex is proposed to forward relevant information from neocortical regions to the hippocampal formation, where a memory trace is rapidly encoded (Danieli et al., 2023;Takehara-Nishiuchi, 2014). Associated regions are reactivated during episodic memory retrieval (Bradley & Sambuco, 2022;Rugg & Vilberg, 2013), and the integrity of the neural connections between regions is integral for the recall of object, spatial, and temporal information (Hayes et al., 2004). Emotionally salient events demonstrate higher activation of the amygdala and corresponding neural circuits, leading to better memory formation (Hammack et al., 2023). Semantic memory recruits many of the same neural circuits used in episodic memory (Tanguay et al., 2023), and the two memory domains interact heavily in memory formation and retrieval (Weidemann et al., 2019). The lateral occipital cortex has additionally been found to help integrate new episodic memories into existing semantic knowledge (Guo & Yang, 2023;Karanian & Slotnick, 2015). In AD, the entorhinal cortex is one of the first cortical regions affected, associated with worse memory retrieval (Rowan et al., 2023), and the inferior temporal lobes are affected soon after (Scheff et al., 2011). This is consistent with findings of initial memory deficits at the transition from preclinical to symptomatic AD. TI is an SUVR weighted composite score derived from regions of interest sensitive to preclinical AD tau pathology (entorhinal cortex, amygdala, inferior temporal cortex, and lateral occipital cortex). Consequentially, 25% of the brain structures comprising this measure have been robustly linked to general memory processing (i.e., the entorhinal cortex), with some additional involvement from the remaining structures. TI is, therefore, particularly sensitive to changes in our composites that feature episodic and semantic memory due to the specific evaluation of tau burden in these brain regions.

Alternatively, TSS is particularly sensitive to deficits in the attention/processing speed and Knight PACC composites. Some studies have demonstrated that amyloid pathology is associated with executive function independent of tau pathology (Tideman et al., 2022), which may influence this finding. However, previous work (Doering et al., 2024) showed TI is more strongly associated with amyloid burden compared with TSS, so underlying amyloid burden is likely not driving this effect. We confirmed this finding by rerunning the model comparison for the attention/processing speed composite with amyloid Centiloid (Klunk et al., 2015;Su et al., 2019) as a covariate and found no change in results (Supplemental Table 8). Attention interacts heavily with several neural processes, modulating distributed neurocognitive systems through inhibition and arousal (Coull, 1998). Cognitive demand of these functions is integrated and evaluated within large-scale multimodal networks, activating bilateral dorsolateral prefrontal and parietal cortices (Corbetta & Shulman, 2002;Kim et al., 2017;Pessoa et al., 2003). The dorsal attention network, attributed to top–down attentional control focusing on goal-oriented selection to attended stimuli, encompasses the superior parietal lobe, intraparietal sulcus, and frontal eye field. The ventral attention network, attributed to bottom–up attentional control focusing on the environmentally driven detection of stimuli, encompasses partially right-lateralized temporoparietal junction, the middle frontal gyrus, and the inferior frontal gyrus (Nani et al., 2019;Vossel et al., 2014). Depending on cognitive task and attended stimuli, additional brain regions are recruited from the frontal, parietal, and sensory-association cortices (Alves et al., 2022;Coull, 1998). In AD, deficits in attention commonly occur after episodic memory impairment is already observed (Malhotra, 2019;Perry, 1999). A prominent theory for this progression in AD is that these attention deficits arise from the spread of early tau pathology from the medial temporal lobe to the parietal lobe (Perry, 1999). Previous studies have shown AD patients with parietal lobe lesions demonstrate impairment in selective attention (Parasuraman & Haxby, 1993;Perry, 1999). Some attention deficits have been found as early as preclinical AD (Bäckman et al., 2005;Balota et al., 2010), which corresponds with preclinical AD spatiotemporal distribution of tau pathology most prevalent in the temporal and parietal lobes (Insel et al., 2023). TI focally evaluates key regions with early AD tau pathology; however, the parietal and frontal lobes are not included in these regions. TSS can capture the spread of tau pathology into the parietal and frontal lobes and thus better captures the neural correlates for attention. TSS is, therefore, particularly sensitive to attention/processing speed impairment because the metric evaluates tau spread across all cortical regions.

The Knight PACC is derived from neuropsychological tests included in the domain-specific composites for episodic memory, semantic memory, and attention/processing speed. However, these domains are not equally represented in the Knight PACC since two of the four neuropsychological tests come from the attention/processing speed composite. Attentional control has been identified as one of the earliest changes in AD (Aschenbrenner et al., 2015;Balota et al., 2010) and hypothesized to drive early memory impairment. The Knight PACC is particularly sensitive to early cognitive change, exemplified with the high representation of attentional tasks in the calculation of the composite score. The sensitivity of TSS to Knight PACC score may be due to particular impairment in attention/processing speed.

We found no substantial relationship between tau pathology and working memory. Previous work (McKay et al., 2024) found the working memory composite consistently demonstrated weak or nonexistent relationships with biomarkers for age and AD pathology. This study supports our findings and suggests the working memory composite is simply not sensitive enough or specific to any early AD-related deficits in working memory, or that there is domain-specific preservation of cognition in early AD. Previous studies have identified impairment in AD patients for various working memory tasks; however, most findings demonstrate mixed results or only mild impairment (Belleville et al., 1996;Kumar et al., 2017;R. G. Morris & Baddeley, 1988). Working memory, similar to attention, is believed to be a multimodal system recruiting large-scale cortical networks spanning across the neocortex (R. G. Morris, 1994). A breakdown in the connections between cortical regions may, therefore, explain deficits observed in AD. Additionally, based on the Working Memory Model (A. Baddeley, 1996;A. D. Baddeley & Hitch, 1974), it has been proposed that initial AD-related working memory deficits arise from the breakdown of a central executive process tasked with regulating attention. Central executive dysfunction thus explains impairment in switching between or coordinating multiple tasks in AD (Germano & Kinsella, 2005;Huntley & Howard, 2010;R. G. Morris, 1994). Working memory and attention have additionally been proposed to share a common underlying executive attention component (McCabe et al., 2010). Given the similarities between attention and working memory, as well as the observed sensitivity of the attention/processing speed composite to preclinical AD impairments, the insignificant findings for the working memory composite are more likely due to measurement properties of the composite itself rather than a sparing of working memory in AD. Future work should be conducted to evaluate the specific working memory neuropsychological tests and further refine the working memory composite in order to increase sensitivity to AD-related working memory impairment.

Despite the specialization of TI and TSS in relation to baseline cognitive impairments, we find TI to better estimate longitudinal cognitive performance and clinical progression than TSS in all measures we tested. This is unexpected for the attention/processing speed composite since TSS better estimates attention deficits at baseline. This finding suggests that TI has better longitudinal measurement properties than TSS for clinical and cognitive outcomes. For example, the binary classification of abnormal voxels in the calculation of TSS can introduce noise to the metric due to false positives. In baseline analyses, the benefits of TSS, such as the ability to capture region-specific tau pathology associated with attention/processing speed, may outweigh the negative impact of the added noise. However, longitudinal analyses introduce additional sources of noise and increase intersubject variability since many factors can influence the rate of cognitive decline. In this case, TI may provide a more homogeneous but clear signal for predicting longitudinal outcomes. Alternatively, this finding could potentially be explained by a secondary wave of attention deficits after the preclinical parietal-related impairment. Previous studies have demonstrated deficits in attention and executive function which strongly recruit frontal lobe regions before the onset of language or visuospatial impairment (Perry, 1999). However, the frontal lobe is relatively spared in AD (Gordon et al., 2018;Lam et al., 2013). This could indicate that attention deficits observed at this stage stem from a breakdown in the neural network, supporting the theory that AD is a disconnection syndrome of disrupted communication between neocortical regions (Delbeuck et al., 2003;Parasuraman & Haxby, 1993;Perry, 1999). Neuropathological studies of AD demonstrate cell-type specific disruption of corticocortical connections, such as the large tract connecting the parietal and frontal cortices (Li et al., 2019;Terry et al., 1991). Secondary deficits in attention at this stage may, therefore, not result from the spread of tau pathology into new regions, but instead from the ongoing accumulation of tau pathology in impacted regions such as the parietal lobe, which substantially damages corticocortical pathways to the frontal lobe over time. TI specifically measures tau pathology within early regions and, therefore, ongoing tau accumulation, whereas TSS is more susceptible to variability due to the larger scope of regions assessed. TI would, therefore, be more sensitive to longitudinal cognitive decline, including impairment in attention.

It is important to address the limitations of the study. First, longitudinal analyses are restricted to cognitive testing as insufficient follow-up tau PET data are available for cognitively impaired participants. Tau PET is relatively new and will continue to be collected in the study cohort. As more data become available, future analyses will evaluate domain-specific cognitive decline in relation to longitudinal tau spread. Second, the participant cohort is mostly white and highly educated, and is thus not representative of the broader population. Lastly, TSS demonstrates variability in older controls, potentially due to voxel-specific false positives. Future analyses should be conducted to evaluate longitudinal voxel-wise stability for participants across AD stages.

Despite these limitations, the study demonstrates several strengths. First, many participants in the study cohort are classified as having preclinical AD, a key stage in the disease in which tau pathology begins to develop. Symptoms are often observed soon after tau pathology onset, resulting in a short period of time to acquire tau PET in this critical window. Unlike many studies focusing on symptomatic AD patients, this study evaluates many preclinical AD participants and can assess differences in tau burden and tau spread in relation to early cognitive impairments. Second, many participants are regularly assessed with neuropsychological and clinical testing in this study, resulting in a rich longitudinal cognitive dataset. Finally, the most prominent strength of this study is the method of quantifying both tau burden and tau spread to evaluate each tau component in tandem and in parallel to better understand early AD tau pathological progression.

In conclusion, our findings support the value in quantification of both TI and TSS from tau PET for evaluating AD progression and related cognitive deficits. TI is a strong biomarker associated with episodic and semantic memory impairment typically seen at early stages of amnestic AD and associated with future general cognitive decline. TSS is better able to capture nonamnestic cognitive deficits such as attention/processing speed, beginning at the preclinical stage, due to the inclusion of all brain regions in its calculation. These findings implicate early focal tau burden for initial memory impairments and implicate early diffuse tau spread for network dysfunction and subsequent impairment in attention. The distinction between these features of tau pathology in relation to cognition may allow for quicker clinical identification of preclinical AD cognitive symptoms, resulting in quicker pathological screening, diagnosis, and better-informed therapeutic routes for treatment. Evaluation of both tau burden and tau spread may additionally be helpful for assessment in clinical trials of tau therapy.

## Supplementary Material

Supplementary Material

## Data Availability

The data used in these analyses are available on request for Knight ADRC athttps://knightadrc.wustl.edu/data-request-form/and for DIAN athttps://dian.wustl.edu/our-research/for-investigators/dian-observational-study-investigator-resources/data-request-form/.

## References

[b1] Alladi , S. , Xuereb , J. , Bak , T. , Nestor , P. , Knibb , J. , Patterson , K. , & Hodges , J. R. ( 2007 ). Focal cortical presentations of Alzheimer’s disease . Brain , 130 ( 10 ), 2636 – 2645 . 10.1093/brain/awm213 17898010

[b2] Alves , P. N. , Forkel , S. J. , Corbetta , M. , & Thiebaut De Schotten , M. ( 2022 ). The subcortical and neurochemical organization of the ventral and dorsal attention networks . Communications Biology , 5 ( 1 ), 1343 . 10.1038/s42003-022-04281-0 36477440 PMC9729227

[b3] Armitage , S. G. ( 1946 ). An analysis of certain psychological tests used for the evaluation of brain injury . Psychological Monographs , 60 ( 1 ), i – 48 . 10.1037/h0093567

[b4] Aschenbrenner , A. J. , Balota , D. A. , Fagan , A. M. , Duchek , J. M. , Benzinger , T. L. S. , & Morris , J. C. ( 2015 ). Alzheimer disease cerebrospinal fluid biomarkers moderate baseline differences and predict longitudinal change in attentional control and episodic memory composites in the adult children study . Journal of the International Neuropsychological Society , 21 ( 8 ), 573 – 583 . 10.1017/S1355617715000776 26416094 PMC4610253

[b5] Bäckman , L. , Jones , S. , Berger , A.-K. , Laukka , E. J. , & Small , B. J. ( 2005 ). Cognitive impairment in preclinical Alzheimer’s disease: A meta-analysis . Neuropsychology , 19 ( 4 ), 520 – 531 . 10.1037/0894-4105.19.4.520 16060827

[b6] Baddeley , A. ( 1996 ). Exploring the central executive . The Quarterly Journal of Experimental Psychology Section A , 49 ( 1 ), 5 – 28 . 10.1080/713755608

[b7] Baddeley , A. D. , & Hitch , G. ( 1974 ). Working memory . Psychology of Learning and Motivation , 8 , 47 – 89 . 10.1016/S0079-7421(08)60452-1

[b8] Balota , D. A. , Tse , C.-S. , Hutchison , K. A. , Spieler , D. H. , Duchek , J. M. , & Morris , J. C. ( 2010 ). Predicting conversion to dementia of the Alzheimer’s type in a healthy control sample: The power of errors in stroop color naming . Psychology and Aging , 25 ( 1 ), 208 – 218 . 10.1037/a0017474 20230140 PMC2886285

[b9] Beekly , D. L. , Ramos , E. M. , Lee , W. W. , Deitrich , W. D. , Jacka , M. E. , Wu , J. , Hubbard , J. L. , Koepsell , T. D. , Morris , J. C. , & Kukull , W. A. ( 2007 ). The National Alzheimer’s Coordinating Center (NACC) Database: The Uniform Data Set . Alzheimer Disease & Associated Disorders , 21 ( 3 ), 249 – 258 . 10.1097/WAD.0b013e318142774e 17804958

[b10] Bejanin , A. , Schonhaut , D. R. , La Joie , R. , Kramer , J. H. , Baker , S. L. , Sosa , N. , Ayakta , N. , Cantwell , A. , Janabi , M. , Lauriola , M. , O’Neil , J. P. , Gorno-Tempini , M. L. , Miller , Z. A. , Rosen , H. J. , Miller , B. L. , Jagust , W. J. , & Rabinovici , G. D. ( 2017 ). Tau pathology and neurodegeneration contribute to cognitive impairment in Alzheimer’s disease . Brain , 140 ( 12 ), 3286 – 3300 . 10.1093/brain/awx243 29053874 PMC5841139

[b11] Belleville , S. , Peretz , I. , & Malenfant , D. ( 1996 ). Examination of the working memory components in normal aging and in dementia of the Alzheimer type . Neuropsychologia , 34 ( 3 ), 195 – 207 . 10.1016/0028-3932(95)00097-6 8868277

[b12] Bloom , G. S. ( 2014 ). Amyloid-β and Tau: The trigger and bullet in Alzheimer disease pathogenesis . JAMA Neurology , 71 ( 4 ), 505 . 10.1001/jamaneurol.2013.5847 24493463 PMC12908160

[b13] Bocancea , D. I. , Svenningsson , A. L. , Van Loenhoud , A. C. , Groot , C. , Barkhof , F. , Strandberg , O. , Smith , R. , for the Alzheimer’s Disease Neuroimaging Initiative , Weiner , M. W. , Aisen , P. , Petersen , R. , Aisen , P. , Petersen , R. , Jack , C. R. , Jagust , W. , Trojanowki , J. Q. , Toga , A. W. , Beckett , L. , Green , R. C. , … Ossenkoppele , R. ( 2023 ). Determinants of cognitive and brain resilience to tau pathology: A longitudinal analysis . Brain , 146 ( 9 ), 3719 – 3734 . 10.1093/brain/awad100 36967222 PMC10473572

[b14] Braak , H. , & Braak , E. ( 1991 ). Neuropathological stageing of Alzheimer-related changes . Acta Neuropathologica , 82 ( 4 ), 239 – 259 . 10.1007/BF00308809 1759558

[b15] Bradley , M. M. , & Sambuco , N. ( 2022 ). Emotional memory and amygdala activation . Frontiers in Behavioral Neuroscience , 16 , 896285 . 10.3389/fnbeh.2022.896285 35769628 PMC9234481

[b16] Brier , M. R. , Gordon , B. , Friedrichsen , K. , McCarthy , J. , Stern , A. , Christensen , J. , Owen , C. , Aldea , P. , Su , Y. , Hassenstab , J. , Cairns , N. J. , Holtzman , D. M. , Fagan , A. M. , Morris , J. C. , Benzinger , T. L. S. , & Ances , B. M. ( 2016 ). Tau and Aβ imaging, CSF measures, and cognition in Alzheimer’s disease . Science Translational Medicine , 8 , 338ra66 . 10.1126/scitranslmed.aaf2362 PMC5267531 27169802

[b17] Brookmeyer , R. , Abdalla , N. , Kawas , C. H. , & Corrada , M. M. ( 2018 ). Forecasting the prevalence of preclinical and clinical Alzheimer’s disease in the United States . Alzheimer’s & Dementia , 14 ( 2 ), 121 – 129 . 10.1016/j.jalz.2017.10.009 PMC5803316 29233480

[b18] Burnham , K. P. , & Anderson , D. R. ( 2004 ). Multimodel inference: Understanding AIC and BIC in model selection . Sociological Methods & Research , 33 ( 2 ), 261 – 304 . 10.1177/0049124104268644

[b19] Cho , H. , Choi , J. Y. , Hwang , M. S. , Kim , Y. J. , Lee , H. M. , Lee , H. S. , Lee , J. H. , Ryu , Y. H. , Lee , M. S. , & Lyoo , C. H. ( 2016 ). In vivo cortical spreading pattern of tau and amyloid in the Alzheimer disease spectrum: Tau and Amyloid in AD . Annals of Neurology , 80 ( 2 ), 247 – 258 . 10.1002/ana.24711 27323247

[b20] Congdon , E. E. , & Sigurdsson , E. M. ( 2018 ). Tau-targeting therapies for Alzheimer disease . Nature Reviews Neurology , 14 ( 7 ), 399 – 415 . 10.1038/s41582-018-0013-z 29895964 PMC6463489

[b21] Corbetta , M. , & Shulman , G. L. ( 2002 ). Control of goal-directed and stimulus-driven attention in the brain . Nature Reviews Neuroscience , 3 ( 3 ), 201 – 215 . 10.1038/nrn755 11994752

[b22] Coull , J. T. ( 1998 ). Neural correlates of attention and arousal: Insights from electrophysiology, functional neuroimaging and psychopharmacology . Progress in Neurobiology , 55 ( 4 ), 343 – 361 . 10.1016/S0301-0082(98)00011-2 9654384

[b23] Craft , S. , Newcomer , J. , Kanne , S. , Dagogo-Jack , S. , Cryer , P. , Sheline , Y. , Luby , J. , Dagogo-Jack , A. , & Alderson , A. ( 1996 ). Memory improvement following induced hyperinsulinemia in Alzheimer’s disease . Neurobiology of Aging , 17 ( 1 ), 123 – 130 . 10.1016/0197-4580(95)02002-0 8786794

[b24] Crutch , S. J. , Lehmann , M. , Schott , J. M. , Rabinovici , G. D. , Rossor , M. N. , & Fox , N. C. ( 2012 ). Posterior cortical atrophy . The Lancet Neurology , 11 ( 2 ), 170 – 178 . 10.1016/S1474-4422(11)70289-7 22265212 PMC3740271

[b25] Danieli , K. , Guyon , A. , & Bethus , I. ( 2023 ). Episodic memory formation: A review of complex Hippocampus input pathways . Progress in Neuro-Psychopharmacology and Biological Psychiatry , 126 , 110757 . 10.1016/j.pnpbp.2023.110757 37086812

[b26] Delbeuck , X. , Van der Linden , M. , & Collette , F. ( 2003 ). Alzheimer’s disease as a disconnection syndrome? Neuropsychology Review , 13 ( 2 ), 79 – 92 . 10.1023/a:1023832305702 12887040

[b27] Doering , S. , McCullough , A. , Gordon , B. A. , Chen , C. D. , McKay , N. , Hobbs , D. , Keefe , S. , Flores , S. , Scott , J. , Smith , H. , Jarman , S. , Jackson , K. , Hornbeck , R. C. , Ances , B. M. , Xiong , C. , Aschenbrenner , A. J. , Hassenstab , J. , Cruchaga , C. , Daniels , A. , … Benzinger , T. L. S. ( 2024 ). Deconstructing pathological tau by biological process in early stages of Alzheimer disease: A method for quantifying tau spatial spread in neuroimaging . eBioMedicine , 103 , 105080 . 10.1016/j.ebiom.2024.105080 38552342 PMC10995809

[b28] Duara , R. , & Barker , W. ( 2022 ). Heterogeneity in Alzheimer’s disease diagnosis and progression rates: Implications for therapeutic trials . Neurotherapeutics , 19 ( 1 ), 8 – 25 . 10.1007/s13311-022-01185-z 35084721 PMC9130395

[b29] Fischl , B. ( 2012 ). FreeSurfer . NeuroImage , 62 ( 2 ), 774 – 781 . 10.1016/j.neuroimage.2012.01.021 22248573 PMC3685476

[b30] Gallagher , M. , & Koh , M. T. ( 2011 ). Episodic memory on the path to Alzheimer’s disease . Current Opinion in Neurobiology , 21 ( 6 ), 929 – 934 . 10.1016/j.conb.2011.10.021 22079495 PMC3254732

[b31] Germano , C. , & Kinsella , G. J. ( 2005 ). Working memory and learning in early Alzheimer’s disease . Neuropsychology Review , 15 ( 1 ), 1 – 10 . 10.1007/s11065-005-3583-7 15929495

[b32] Gollan , T. H. , Garcia , D. L. , Stasenko , A. , Murillo , M. , Kim , C. , Galasko , D. , & Salmon , D. P. ( 2024 ). The MINT Sprint 2.0: A picture naming test for detection of naming impairments in Alzheimer’s disease and in preclinical AD . Alzheimer’s & Dementia , 20 ( 1 ), 112 – 123 . 10.1002/alz.13381 PMC10916946 37464962

[b33] Goodglass , H. , & Kaplan , E. ( 1983 ). Boston diagnostic aphasia examination booklet . Lea & Febiger .

[b34] Gordon , B. A. , Friedrichsen , K. , Brier , M. , Blazey , T. , Su , Y. , Christensen , J. , Aldea , P. , McConathy , J. , Holtzman , D. M. , Cairns , N. J. , Morris , J. C. , Fagan , A. M. , Ances , B. M. , & Benzinger , T. L. S. ( 2016 ). The relationship between cerebrospinal fluid markers of Alzheimer pathology and positron emission tomography tau imaging . Brain , 139 ( 8 ), 2249 – 2260 . 10.1093/brain/aww139 27286736 PMC4958902

[b35] Gordon , B. A. , McCullough , A. , Mishra , S. , Blazey , T. M. , Su , Y. , Christensen , J. , Dincer , A. , Jackson , K. , Hornbeck , R. C. , Morris , J. C. , Ances , B. M. , & Benzinger , T. L. S. ( 2018 ). Cross‐sectional and longitudinal atrophy is preferentially associated with tau rather than amyloid β positron emission tomography pathology . Alzheimer’s & Dementia: Diagnosis, Assessment & Disease Monitoring , 10 ( 1 ), 245 – 252 . 10.1016/j.dadm.2018.02.003 PMC5956934 29780869

[b36] Grober , E. , Lipton , R. B. , Katz , M. , & Sliwinski , M. ( 1998 ). Demographic influences on free and cued selective reminding performance in older persons . Journal of Clinical and Experimental Neuropsychology , 20 ( 2 ), 221 – 226 . 10.1076/jcen.20.2.221.1177 9777476

[b37] Gulisano , W. , Maugeri , D. , Baltrons , M. A. , Fà , M. , Amato , A. , Palmeri , A. , D’Adamio , L. , Grassi , C. , Devanand , D. P. , Honig , L. S. , Puzzo , D. , & Arancio , O. ( 2018 ). Role of Amyloid-β and Tau Proteins in Alzheimer’s disease: Confuting the amyloid cascade . Journal of Alzheimer’s Disease , 64 ( s1 ), S611 – S631 . 10.3233/JAD-179935 PMC8371153 29865055

[b38] Guo , D. , & Yang , J. ( 2023 ). Reactivation of schema representation in lateral occipital cortex supports successful memory encoding . Cerebral Cortex , 33 ( 10 ), 5968 – 5980 . 10.1093/cercor/bhac475 36520467

[b39] Hammack , R. J. , Fischer , V. E. , Andrade , M. A. , & Toney , G. M. ( 2023 ). Anterior basolateral amygdala neurons comprise a remote fear memory engram . Frontiers in Neural Circuits , 17 , 1167825 . 10.3389/fncir.2023.1167825 37180762 PMC10174320

[b40] Hayes , S. M. , Ryan , L. , Schnyer , D. M. , & Nadel , L. ( 2004 ). An fMRI study of episodic memory: Retrieval of object, spatial, and temporal information . Behavioral Neuroscience , 118 ( 5 ), 885 – 896 . 10.1037/0735-7044.118.5.885 15506871 PMC3172698

[b41] Hennawy , M. , Sabovich , S. , Liu , C. S. , Herrmann , N. , & Lanctôt , K. L. ( 2019 ). Sleep and attention in Alzheimer’s disease . The Yale Journal of Biology and Medicine , 92 ( 1 ), 53 – 61 . https://pmc.ncbi.nlm.nih.gov/articles/PMC6430169/ 30923473 PMC6430169

[b42] Huff , M. J. , Balota , D. A. , Minear , M. , Aschenbrenner , A. J. , & Duchek , J. M. ( 2015 ). Dissociative global and local task-switching costs across younger adults, middle-aged adults, older adults, and very mild Alzheimer’s disease individuals . Psychology and Aging , 30 ( 4 ), 727 – 739 . 10.1037/pag0000057 26652720 PMC4681312

[b43] Hughes , C. P. , Berg , L. , Danziger , W. , Coben , L. A. , & Martin , R. L. ( 1982 ). A new clinical scale for the staging of dementia . British Journal of Psychiatry , 140 ( 6 ), 566 – 572 . 10.1192/bjp.140.6.566 7104545

[b44] Huntley , J. D. , & Howard , R. J. ( 2010 ). Working memory in early Alzheimer’s disease: A neuropsychological review . International Journal of Geriatric Psychiatry , 25 ( 2 ), 121 – 132 . 10.1002/gps.2314 19672843

[b45] Insel , P. S. , Young , C. B. , Aisen , P. S. , Johnson , K. A. , Sperling , R. A. , Mormino , E. C. , & Donohue , M. C. ( 2023 ). Tau positron emission tomography in preclinical Alzheimer’s disease . Brain , 146 ( 2 ), 700 – 711 . 10.1093/brain/awac299 35962782 PMC10169284

[b46] Ivanova , I. , Salmon , D. P. , & Gollan , T. H. ( 2013 ). The multilingual naming test in Alzheimer’s disease: Clues to the origin of naming impairments . Journal of the International Neuropsychological Society , 19 ( 3 ), 272 – 283 . 10.1017/S1355617712001282 23298442 PMC4356120

[b47] Jack , C. R. , Bennett , D. A. , Blennow , K. , Carrillo , M. C. , Dunn , B. , Haeberlein , S. B. , Holtzman , D. M. , Jagust , W. , Jessen , F. , Karlawish , J. , Liu , E. , Molinuevo , J. L. , Montine , T. , Phelps , C. , Rankin , K. P. , Rowe , C. C. , Scheltens , P. , Siemers , E. , Snyder , H. M. , … Silverberg , N. ( 2018 ). NIA‐AA Research Framework: Toward a biological definition of Alzheimer’s disease . Alzheimer’s & Dementia , 14 ( 4 ), 535 – 562 . 10.1016/j.jalz.2018.02.018 PMC5958625 29653606

[b48] Jack , C. R. , Wiste , H. J. , Weigand , S. D. , Therneau , T. M. , Lowe , V. J. , Knopman , D. S. , Gunter , J. L. , Senjem , M. L. , Jones , D. T. , Kantarci , K. , Machulda , M. M. , Mielke , M. M. , Roberts , R. O. , Vemuri , P. , Reyes , D. A. , & Petersen , R. C. ( 2017 ). Defining imaging biomarker cut points for brain aging and Alzheimer’s disease . Alzheimer’s & Dementia , 13 ( 3 ), 205 – 216 . 10.1016/j.jalz.2016.08.005 PMC5344738 27697430

[b49] Jellinger , K. A. ( 2022 ). Recent update on the heterogeneity of the Alzheimer’s disease spectrum . Journal of Neural Transmission , 129 ( 1 ), 1 – 24 . 10.1007/s00702-021-02449-2 34919190

[b50] Johnson , J. K. , Head , E. , Kim , R. , Starr , A. , & Cotman , C. W. ( 1999 ). Clinical and pathological evidence for a frontal variant of Alzheimer disease . Archives of Neurology , 56 ( 10 ), 1233 . 10.1001/archneur.56.10.1233 10520939

[b51] Karanian , J. M. , & Slotnick , S. D. ( 2015 ). Memory for shape reactivates the lateral occipital complex . Brain Research , 1603 , 124 – 132 . 10.1016/j.brainres.2015.01.024 25623846

[b52] Kim , N. Y. , Wittenberg , E. , & Nam , C. S. ( 2017 ). Behavioral and neural correlates of executive function: Interplay between inhibition and updating processes . Frontiers in Neuroscience , 11 , 378 . 10.3389/fnins.2017.00378 28713237 PMC5492464

[b53] Klunk , W. E. , Koeppe , R. A. , Price , J. C. , Benzinger , T. L. , Devous , M. D. , Jagust , W. J. , Johnson , K. A. , Mathis , C. A. , Minhas , D. , Pontecorvo , M. J. , Rowe , C. C. , Skovronsky , D. M. , & Mintun , M. A. ( 2015 ). The Centiloid Project: Standardizing quantitative amyloid plaque estimation by PET . Alzheimer’s & Dementia , 11 ( 1 ), 1 . 10.1016/j.jalz.2014.07.003 PMC4300247 25443857

[b54] Kumar , S. , Zomorrodi , R. , Ghazala , Z. , Goodman , M. S. , Blumberger , D. M. , Cheam , A. , Fischer , C. , Daskalakis , Z. J. , Mulsant , B. H. , Pollock , B. G. , & Rajji , T. K. ( 2017 ). Extent of dorsolateral prefrontal cortex plasticity and its association with working memory in patients with Alzheimer disease . JAMA Psychiatry , 74 ( 12 ), 1266 . 10.1001/jamapsychiatry.2017.3292 29071355 PMC6583382

[b55] La Joie , R. , Visani , A. V. , Baker , S. L. , Brown , J. A. , Bourakova , V. , Cha , J. , Chaudhary , K. , Edwards , L. , Iaccarino , L. , Janabi , M. , Lesman-Segev , O. H. , Miller , Z. A. , Perry , D. C. , O’Neil , J. P. , Pham , J. , Rojas , J. C. , Rosen , H. J. , Seeley , W. W. , Tsai , R. M. , … Rabinovici , G. D. ( 2020 ). Prospective longitudinal atrophy in Alzheimer’s disease correlates with the intensity and topography of baseline tau-PET . Science Translational Medicine , 12 ( 524 ), eaau5732 . 10.1126/scitranslmed.aau5732 31894103 PMC7035952

[b56] Lam , B. , Masellis , M. , Freedman , M. , Stuss , D. T. , & Black , S. E. ( 2013 ). Clinical, imaging, and pathological heterogeneity of the Alzheimer’s disease syndrome . Alzheimer’s Research & Therapy , 5 ( 1 ), 1 . 10.1186/alzrt155 PMC3580331 23302773

[b57] Li , R. , Nguyen , T. , Potter , T. , & Zhang , Y. ( 2019 ). Dynamic cortical connectivity alterations associated with Alzheimer’s disease: An EEG and fNIRS integration study . NeuroImage: Clinical , 21 , 101622 . 10.1016/j.nicl.2018.101622 30527906 PMC6411655

[b58] Malhotra , P. A. ( 2019 ). Impairments of attention in Alzheimer’s disease . Current Opinion in Psychology , 29 , 41 – 48 . 10.1016/j.copsyc.2018.11.002 30496975

[b59] McCabe , D. P. , Roediger , H. L. , McDaniel , M. A. , Balota , D. A. , & Hambrick , D. Z. ( 2010 ). The relationship between working memory capacity and executive functioning: Evidence for a common executive attention construct . Neuropsychology , 24 ( 2 ), 222 – 243 . 10.1037/a0017619 20230116 PMC2852635

[b60] McKay , N. S. , Gordon , B. A. , Hornbeck , R. C. , Dincer , A. , Flores , S. , Keefe , S. J. , Joseph-Mathurin , N. , Jack , C. R. , Koeppe , R. , Millar , P. R. , Ances , B. M. , Chen , C. D. , Daniels , A. , Hobbs , D. A. , Jackson , K. , Koudelis , D. , Massoumzadeh , P. , McCullough , A. , Nickels , M. L. , … La Fougère , C. ( 2023 ). Positron emission tomography and magnetic resonance imaging methods and datasets within the Dominantly Inherited Alzheimer Network (DIAN) . Nature Neuroscience , 26 ( 8 ), 1449 – 1460 . 10.1038/s41593-023-01359-8 37429916 PMC10400428

[b61] McKay , N. S. , Millar , P. R. , Nicosia , J. , Aschenbrenner , A. J. , Gordon , B. A. , Benzinger , T. L. S. , Cruchaga , C. C. , Schindler , S. E. , Morris , J. C. , & Hassenstab , J. ( 2024 ). Pick a PACC: Comparing domain-specific and general cognitive composites in Alzheimer disease research . Neuropsychology , 38 ( 5 ), 443 – 464 . 10.1037/neu0000949 38602816 PMC11176005

[b62] Meyer , A. M. , Snider , S. F. , Campbell , R. E. , & Friedman , R. B. ( 2015 ). Phonological short-term memory in logopenic variant primary progressive aphasia and mild Alzheimer’s disease . Cortex , 71 , 183 – 189 . 10.1016/j.cortex.2015.07.003 26232551 PMC4521400

[b63] Mishra , S. , Gordon , B. A. , Su , Y. , Christensen , J. , Friedrichsen , K. , Jackson , K. , Hornbeck , R. , Balota , D. A. , Cairns , N. J. , Morris , J. C. , Ances , B. M. , & Benzinger , T. L. S. ( 2017 ). AV-1451 PET imaging of tau pathology in preclinical Alzheimer disease: Defining a summary measure . NeuroImage , 161 , 171 – 178 . 10.1016/j.neuroimage.2017.07.050 28756238 PMC5696044

[b64] Monsell , S. E. , Dodge , H. H. , Zhou , X.-H. , Bu , Y. , Besser , L. M. , Mock , C. , Hawes , S. E. , Kukull , W. A. , & Weintraub , S. ( 2016 ). Results from the NACC uniform data set neuropsychological battery crosswalk study . Alzheimer Disease & Associated Disorders , 30 ( 2 ), 134 – 139 . 10.1097/WAD.0000000000000111 26485498 PMC4834278

[b65] Morris , J. C. ( 1993 ). The Clinical Dementia Rating (CDR): Current version and scoring rules . Neurology , 43 ( 11 ), 2412.2 – 2412-a . 10.1212/WNL.43.11.2412-a 8232972

[b66] Morris , J. C. , Weintraub , S. , Chui , H. C. , Cummings , J. , DeCarli , C. , Ferris , S. , Foster , N. L. , Galasko , D. , Graff-Radford , N. , Peskind , E. R. , Beekly , D. , Ramos , E. M. , & Kukull , W. A. ( 2006 ). The Uniform Data Set (UDS): Clinical and cognitive variables and descriptive data from Alzheimer disease centers . Alzheimer Disease & Associated Disorders , 20 ( 4 ), 210 – 216 . 10.1097/01.wad.0000213865.09806.92 17132964

[b67] Morris , R. G. ( 1994 ). Working memory in Alzheimer-type dementia . Neuropsychology , 8 ( 4 ), 544 – 554 . 10.1037/0894-4105.8.4.544

[b68] Morris , R. G. , & Baddeley , A. D. ( 1988 ). Primary and working memory functioning in Alzheimer-type dementia . Journal of Clinical and Experimental Neuropsychology , 10 ( 2 ), 279 – 296 . 10.1080/01688638808408242 3280591

[b69] Moulder , K. L. , Snider , B. , Mills , S. L. , Buckles , V. D. , Santacruz , A. M. , Bateman , R. J. , & Morris , J. C. ( 2013 ). Dominantly Inherited Alzheimer Network: Facilitating research and clinical trials . Alzheimer’s Research & Therapy , 5 ( 5 ), 48 . 10.1186/alzrt213 PMC3978584 24131566

[b70] Nadel , L. , & Moscovitch , M. ( 1997 ). Memory consolidation, retrograde amnesia and the hippocampal complex . Current Opinion in Neurobiology , 7 ( 2 ), 217 – 227 . 10.1016/S0959-4388(97)80010-4 9142752

[b71] Nani , A. , Manuello , J. , Mancuso , L. , Liloia , D. , Costa , T. , & Cauda , F. ( 2019 ). The neural correlates of consciousness and attention: Two sister processes of the brain . Frontiers in Neuroscience , 13 , 1169 . 10.3389/fnins.2019.01169 31749675 PMC6842945

[b72] Nelson , P. T. , Alafuzoff , I. , Bigio , E. H. , Bouras , C. , Braak , H. , Cairns , N. J. , Castellani , R. J. , Crain , B. J. , Davies , P. , Tredici , K. D. , Duyckaerts , C. , Frosch , M. P. , Haroutunian , V. , Hof , P. R. , Hulette , C. M. , Hyman , B. T. , Iwatsubo , T. , Jellinger , K. A. , Jicha , G. A. , … Beach , T. G. ( 2012 ). Correlation of Alzheimer disease neuropathologic changes with cognitive status: A review of the literature . Journal of Neuropathology & Experimental Neurology , 71 ( 5 ), 362 – 381 . 10.1097/NEN.0b013e31825018f7 22487856 PMC3560290

[b73] Nordberg , A. ( 2004 ). PET imaging of amyloid in Alzheimer’s disease . The Lancet Neurology , 3 ( 9 ), 519 – 527 . 10.1016/S1474-4422(04)00853-1 15324720

[b74] Ossenkoppele , R. , Schonhaut , D. R. , Schöll , M. , Lockhart , S. N. , Ayakta , N. , Baker , S. L. , O’Neil , J. P. , Janabi , M. , Lazaris , A. , Cantwell , A. , Vogel , J. , Santos , M. , Miller , Z. A. , Bettcher , B. M. , Vossel , K. A. , Kramer , J. H. , Gorno-Tempini , M. L. , Miller , B. L. , Jagust , W. J. , & Rabinovici , G. D. ( 2016 ). Tau PET patterns mirror clinical and neuroanatomical variability in Alzheimer’s disease . Brain , 139 ( 5 ), 1551 – 1567 . 10.1093/brain/aww027 26962052 PMC5006248

[b75] Ossenkoppele , R. , Smith , R. , Ohlsson , T. , Strandberg , O. , Mattsson , N. , Insel , P. S. , Palmqvist , S. , & Hansson , O. ( 2019 ). Associations between tau, Aβ, and cortical thickness with cognition in Alzheimer disease . Neurology , 92 ( 6 ), e601 – e612 . 10.1212/WNL.0000000000006875 30626656 PMC6382060

[b76] Parasuraman , R. , & Haxby , J. V. ( 1993 ). Attention and brain function in Alzheimer’s disease: A review . Neuropsychology , 7 ( 3 ), 242 – 272 . 10.1037/0894-4105.7.3.242

[b77] Perry , R. J. ( 1999 ). Attention and executive deficits in Alzheimer’s disease: A critical review . Brain , 122 ( 3 ), 383 – 404 . 10.1093/brain/122.3.383 10094249

[b78] Pessoa , L. , Kastner , S. , & Ungerleider , L. G. ( 2003 ). Neuroimaging studies of attention: From modulation of sensory processing to top-down control . The Journal of Neuroscience , 23 ( 10 ), 3990 – 3998 . 10.1523/JNEUROSCI.23-10-03990.2003 12764083 PMC6741071

[b79] Preiß , D. , Billette , O. V. , Schneider , A. , Spotorno , N. , & Nestor , P. J. ( 2019 ). The atrophy pattern in Alzheimer-related PPA is more widespread than that of the frontotemporal lobar degeneration associated variants . NeuroImage: Clinical , 24 , 101994 . 10.1016/j.nicl.2019.101994 31505368 PMC6734177

[b80] Rogers , S. L. , & Friedman , R. B. ( 2008 ). The underlying mechanisms of semantic memory loss in Alzheimer’s disease and semantic dementia . Neuropsychologia , 46 ( 1 ), 12 – 21 . 10.1016/j.neuropsychologia.2007.08.010 17897685 PMC2255584

[b81] Rousset , O. G. , Ma , Y. , & Evans , A. C. ( 1998 ). Correction for partial volume effects in PET: Principle and validation . Journal of Nuclear Medicine: Official Publication, Society of Nuclear Medicine , 39 ( 5 ), 904 – 911 . https://pubmed.ncbi.nlm.nih.gov/9591599/ 9591599

[b82] Rowan , M. , Goettemoeller , A. , Banks , E. , McCann , K. , Kumar , P. , South , K. , Olah , V. , Ramelow , C. , Duong , D. , Seyfried , N. , Rangaraju , S. , & Weinshenker , D. ( 2023 ). Entorhinal cortex vulnerability to human APP expression promotes hyperexcitability and tau pathology . In Review . 10.21203/rs.3.rs-3370607/v1 PMC11387477 39256379

[b83] Rugg , M. D. , & Vilberg , K. L. ( 2013 ). Brain networks underlying episodic memory retrieval . Current Opinion in Neurobiology , 23 ( 2 ), 255 – 260 . 10.1016/j.conb.2012.11.005 23206590 PMC3594562

[b84] Sawyer , R. P. , Rodriguez-Porcel , F. , Hagen , M. , Shatz , R. , & Espay , A. J. ( 2017 ). Diagnosing the frontal variant of Alzheimer’s disease: A clinician’s yellow brick road . Journal of Clinical Movement Disorders , 4 ( 1 ), 2 . 10.1186/s40734-017-0052-4 28265458 PMC5333400

[b85] Scheff , S. W. , Price , D. A. , Schmitt , F. A. , Scheff , M. A. , & Mufson , E. J. ( 2011 ). Synaptic loss in the inferior temporal gyrus in mild cognitive impairment and Alzheimer’s disease . Journal of Alzheimer’s Disease , 24 ( 3 ), 547 – 557 . 10.3233/JAD-2011-101782 PMC3098316 21297265

[b86] Small , B. J. , Mobly , J. L. , Laukka , E. J. , Jones , S. , & Bäckman , L. ( 2003 ). Cognitive deficits in preclinical Alzheimer’s disease: Cognitive deficits in preclinical AD . Acta Neurologica Scandinavica , 107 , 29 – 33 . 10.1034/j.1600-0404.107.s179.6.x 12603248

[b87] Squire , L. R. , & Alvarez , P. ( 1995 ). Retrograde amnesia and memory consolidation: A neurobiological perspective . Current Opinion in Neurobiology , 5 ( 2 ), 169 – 177 . 10.1016/0959-4388(95)80023-9 7620304

[b88] Su , Y. , Blazey , T. M. , Snyder , A. Z. , Raichle , M. E. , Marcus , D. S. , Ances , B. M. , Bateman , R. J. , Cairns , N. J. , Aldea , P. , Cash , L. , Christensen , J. J. , Friedrichsen , K. , Hornbeck , R. C. , Farrar , A. M. , Owen , C. J. , Mayeux , R. , Brickman , A. M. , Klunk , W. , Price , J. C. , … Benzinger , T. L. S. ( 2015 ). Partial volume correction in quantitative amyloid imaging . NeuroImage , 107 , 55 – 64 . 10.1016/j.neuroimage.2014.11.058 25485714 PMC4300252

[b89] Su , Y. , D’Angelo , G. M. , Vlassenko , A. G. , Zhou , G. , Snyder , A. Z. , Marcus , D. S. , Blazey , T. M. , Christensen , J. J. , Vora , S. , Morris , J. C. , Mintun , M. A. , & Benzinger , T. L. S. ( 2013 ). Quantitative analysis of PiB-PET with FreeSurfer ROIs . PLoS One , 8 ( 11 ), e73377 . 10.1371/journal.pone.0073377 24223109 PMC3819320

[b90] Su , Y. , Flores , S. , Wang , G. , Hornbeck , R. C. , Speidel , B. , Joseph‐Mathurin , N. , Vlassenko , A. G. , Gordon , B. A. , Koeppe , R. A. , Klunk , W. E. , Jack , C. R. , Farlow , M. R. , Salloway , S. , Snider , B. J. , Berman , S. B. , Roberson , E. D. , Brosch , J. , Jimenez‐Velazques , I. , Dyck , C. H. , … Benzinger , T. L. S. ( 2019 ). Comparison of Pittsburgh compound B and florbetapir in cross‐sectional and longitudinal studies . Alzheimer’s & Dementia: Diagnosis, Assessment & Disease Monitoring , 11 ( 1 ), 180 – 190 . 10.1016/j.dadm.2018.12.008 PMC6389727 30847382

[b91] Takeda , S. ( 2019 ). Tau propagation as a diagnostic and therapeutic target for dementia: Potentials and unanswered questions . Frontiers in Neuroscience , 13 , 1274 . 10.3389/fnins.2019.01274 31920473 PMC6923174

[b92] Takehara-Nishiuchi , K. ( 2014 ). Entorhinal cortex and consolidated memory . Neuroscience Research , 84 , 27 – 33 . 10.1016/j.neures.2014.02.012 24642278

[b93] Tanguay , A. F. , Palombo , D. J. , Love , B. , Glikstein , R. , Davidson , P. S. , & Renoult , L. ( 2023 ). The shared and unique neural correlates of personal semantic, general semantic, and episodic memory . eLife , 12 , e83645 . 10.7554/eLife.83645 37987578 PMC10662951

[b94] Terry , R. D. , Masliah , E. , Salmon , D. P. , Butters , N. , DeTeresa , R. , Hill , R. , Hansen , L. A. , & Katzman , R. ( 1991 ). Physical basis of cognitive alterations in Alzheimer’s disease: Synapse loss is the major correlate of cognitive impairment . Annals of Neurology , 30 ( 4 ), 572 – 580 . 10.1002/ana.410300410 1789684

[b95] Tideman , P. , Stomrud , E. , Leuzy , A. , Mattsson-Carlgren , N. , Palmqvist , S. , Hansson , O. , & for the Alzheimer’s Disease Neuroimaging Initiative . ( 2022 ). Association of β-amyloid accumulation with executive function in adults with unimpaired cognition . Neurology , 98 ( 15 ), e1525 – e1533 . 10.1212/WNL.0000000000013299 35022305 PMC9012270

[b96] Villemagne , V. L. , Leuzy , A. , Bohorquez , S. S. , Bullich , S. , Shimada , H. , Rowe , C. C. , Bourgeat , P. , Lopresti , B. , Huang , K. , Krishnadas , N. , Fripp , J. , Takado , Y. , Gogola , A. , Minhas , D. , Weimer , R. , Higuchi , M. , Stephens , A. , Hansson , O. , Doré , V. , & Alzheimer’s Disease Neuroimaging Initiative and the AIBL research group . ( 2023 ). CenTauR: Toward a universal scale and masks for standardizing tau imaging studies . Alzheimer’s & Dementia: Diagnosis, Assessment & Disease Monitoring , 15 ( 3 ), e12454 . 10.1002/dad2.12454 PMC10326476 37424964

[b97] Vossel , S. , Geng , J. J. , & Fink , G. R. ( 2014 ). Dorsal and ventral attention systems: Distinct neural circuits but collaborative roles . The Neuroscientist: A Review Journal Bringing Neurobiology, Neurology and Psychiatry , 20 ( 2 ), 150 – 159 . 10.1177/1073858413494269 23835449 PMC4107817

[b98] Wechsler , D. ( 1981 ). The psychometric tradition: Developing the Wechsler adult intelligence scale . Contemporary Educational Psychology , 6 ( 2 ), 82 – 85 . 10.1016/0361-476X(81)90035-7

[b99] Wechsler , D. ( 1997a ). Wechsler Memory Scale ( 3rd ed.). The Psychological Corporation .

[b100] Wechsler , D. ( 1997b ). WMS-III: Wechsler memory scale administration and scoring manual . The Psychological Corporation .

[b101] Weidemann , C. T. , Kragel , J. E. , Lega , B. C. , Worrell , G. A. , Sperling , M. R. , Sharan , A. D. , Jobst , B. C. , Khadjevand , F. , Davis , K. A. , Wanda , P. A. , Kadel , A. , Rizzuto , D. S. , & Kahana , M. J. ( 2019 ). Neural activity reveals interactions between episodic and semantic memory systems during retrieval . Journal of Experimental Psychology: General , 148 ( 1 ), 1 – 12 . 10.1037/xge0000480 30596439 PMC6419095

[b102] Weintraub , S. , Salmon , D. , Mercaldo , N. , Ferris , S. , Graff-Radford , N. R. , Chui , H. , Cummings , J. , DeCarli , C. , Foster , N. L. , Galasko , D. , Peskind , E. , Dietrich , W. , Beekly , D. L. , Kukull , W. A. , & Morris , J. C. ( 2009 ). The Alzheimer’s Disease Centers’ Uniform Data Set (UDS): The neuropsychologic test battery . Alzheimer Disease & Associated Disorders , 23 ( 2 ), 91 – 101 . 10.1097/WAD.0b013e318191c7dd 19474567 PMC2743984

[b103] Zhang , H. , Cao , Y. , Ma , L. , Wei , Y. , & Li , H. ( 2021 ). Possible mechanisms of Tau Spread and toxicity in Alzheimer’s disease . Frontiers in Cell and Developmental Biology , 9 , 707268 . 10.3389/fcell.2021.707268 34395435 PMC8355602

