# Supplementary material for: Domain-specific cognitive impairment is differentially affected by Alzheimer disease tau pathologic burden and spread
Source: Imaging Neurosci (Camb). 2024 Dec 19;2:imag-2-00405. doi: 10.1162/imag_a_00405 (PMC12176422; doi:10.1162/imag_a_00405)
Supplement: Supplementary Material [file imag_a_00405-supp.pdf]

**Supplemental Material**

**Supplemental Figure 1. Identification of “Low” and “High” Values for TI and TSS.**

Gaussian mixture models were conducted separately for participant baseline TI and TSS with two underlying distributions. Distribution means were identified as representative “low” or “high” values for TI and TSS in the study cohort.

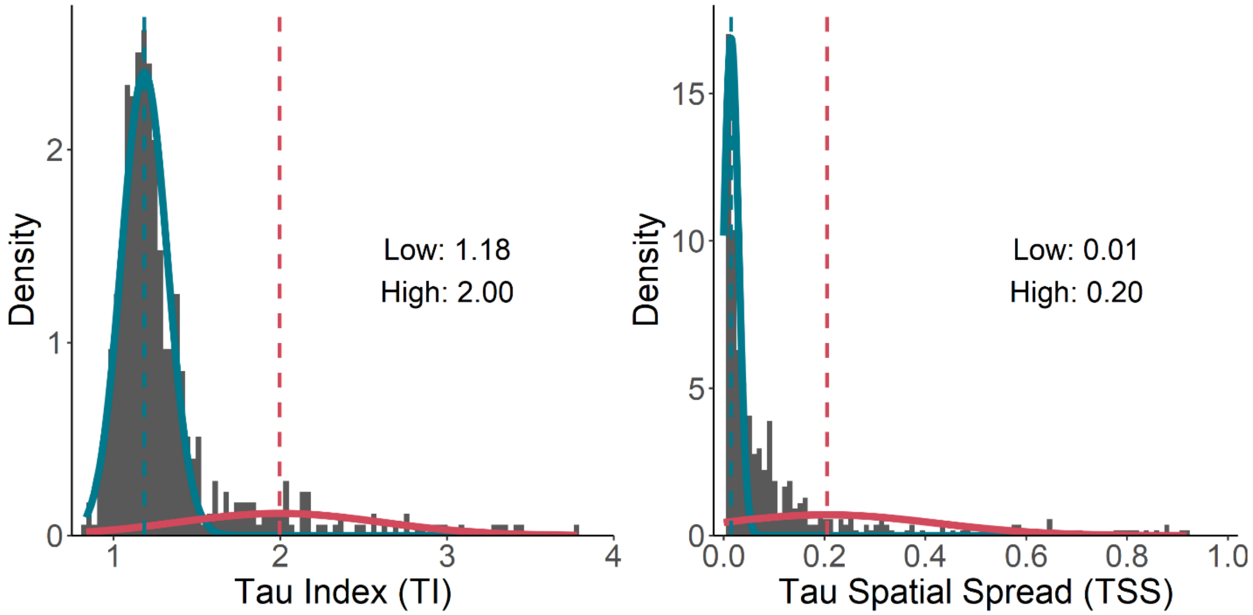

**Supplemental Figure 2. Distribution of TI and TSS for Younger Controls and Disease Stage Groups.**

Distribution summaries of Tau Index and Tau Spatial Spread for younger control, older control, Preclinical AD, and Symptomatic AD groups. Mean, standard deviation (SD), median, interquartile range (IQR), minimum, and maximum are reported for each group.

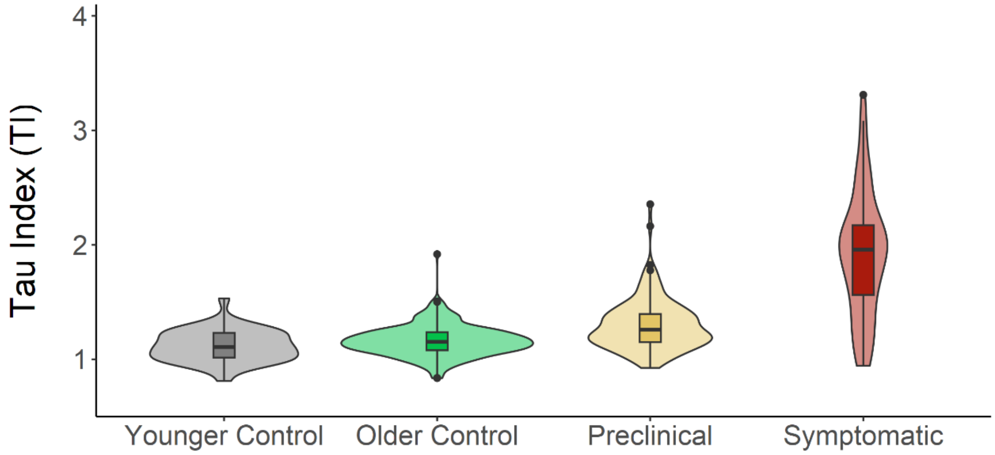


| | Younger Control | Older Control | Preclinical | Symptomatic |
|---|---|---|---|---|
| Mean | 1.12 | 1.16 | 1.30 | 1.92 |
| SD | 0.14 | 0.13 | 0.22 | 0.54 |
| Median | 1.11 | 1.15 | 1.26 | 1.96 |
| IQR | 0.22 | 0.16 | 0.25 | 0.61 |
| Min | 0.81 | 0.84 | 0.92 | 0.94 |
| Max | 1.53 | 1.92 | 2.35 | 3.31 |

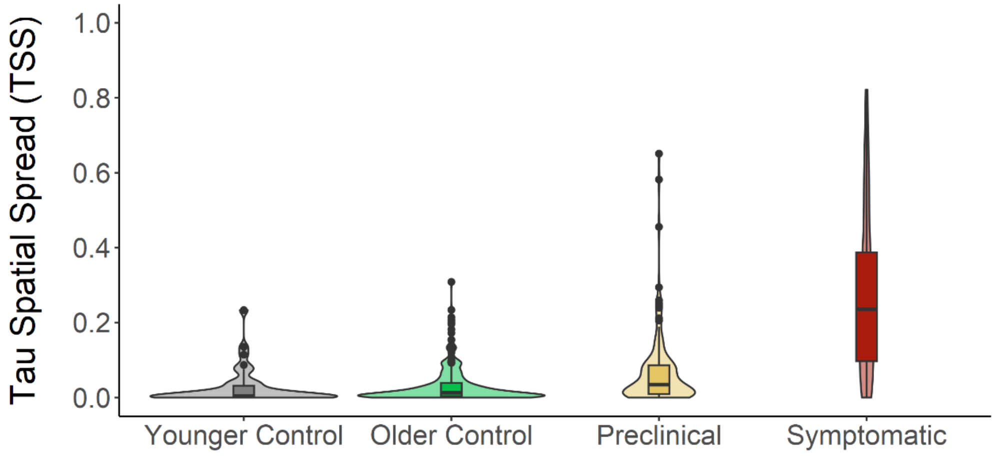


| | Younger Control | Older Control | Preclinical | Symptomatic |
|---|---|---|---|---|
| Mean | 0.03 | 0.03 | 0.07 | 0.28 |
| SD | 0.05 | 0.05 | 0.10 | 0.22 |
| Median | 0.00 | 0.01 | 0.03 | 0.24 |
| IQR | 0.03 | 0.04 | 0.08 | 0.29 |
| Min | 0.00 | 0.00 | 0.00 | 0.00 |
| Max | 0.23 | 0.31 | 0.65 | 0.82 |

**Supplemental Table 1. Cognitive Domain Composite Scores vs. Disease Stage Groups.**

| | Group 1 | Group 2 | W | p (raw) | p (adjusted) | Estimate | Lower CL | Upper CL | Z | Effect Size |
|---|---|---|---|---|---|---|---|---|---|---|
| **PACC** | OC | Preclinical | 19221 | 0.98 | 0.98 | -0.0018 | -0.15 | 0.15 | -0.024 | 0.0012 |
| | Preclinical | Symptomatic | 6619 | <0.0001 | **<0.0001** | 1.77 | 1.45 | 2.10 | 8.79 | 0.64 |
| | OC | Symptomatic | 13658 | <0.0001 | **<0.0001** | 1.78 | 1.49 | 2.08 | 9.63 | 0.53 |
| **Episodic** | OC | Preclinical | 8106 | 0.72 | 0.72 | -0.038 | -0.24 | 0.17 | -0.35 | 0.021 |
| | Preclinical | Symptomatic | 4469 | <0.0001 | **<0.0001** | 2.07 | 1.78 | 2.36 | 8.71 | 0.73 |
| | OC | Symptomatic | 9073.5 | <0.0001 | **<0.0001** | 2.03 | 1.74 | 2.31 | 9.44 | 0.61 |
| **Semantic** | OC | Preclinical | 8368 | 0.88 | 0.88 | -0.015 | -0.19 | 0.16 | -0.15 | 0.0091 |
| | Preclinical | Symptomatic | 3985 | <0.0001 | **<0.0001** | 1.13 | 0.76 | 1.50 | 5.99 | 0.50 |
| | OC | Symptomatic | 8149 | <0.0001 | **<0.0001** | 1.10 | 0.78 | 1.44 | 6.68 | 0.43 |
| **Working** | OC | Preclinical | 8482 | 0.98 | 0.98 | -0.0000086 | -0.21 | 0.22 | 0.03 | 0.0018 |
| | Preclinical | Symptomatic | 3936.5 | <0.0001 | **<0.0001** | 0.85 | 0.59 | 1.13 | 5.79 | 0.48 |
| | OC | Symptomatic | 8022.5 | <0.0001 | **<0.0001** | 0.87 | 0.62 | 1.11 | 6.40 | 0.41 |
| **Attention** | OC | Preclinical | 19454 | 0.55 | 0.55 | 0.041 | -0.090 | 0.18 | 0.60 | 0.030 |
| | Preclinical | Symptomatic | 6295 | <0.0001 | **<0.0001** | 1.85 | 1.41 | 2.28 | 7.67 | 0.56 |
| | OC | Symptomatic | 12961 | <0.0001 | **<0.0001** | 1.88 | 1.50 | 2.31 | 8.59 | 0.47 |

Comparison between disease-stage groups for each cognitive domain composite. Pairwise comparisons conducted with Wilcoxon-Mann-Whitney U tests. P-values reported before (raw) and after (adjusted) multiple comparison correction using Benjamini-Hochberg procedure.

**Supplemental Table 2. Tau Metrics vs. Disease Stage Groups.**

| | Group 1 | Group 2 | W | p (raw) | p (adjusted) | Estimate | Lower CL | Upper CL | Z | Effect Size |
|---|---|---|---|---|---|---|---|---|---|---|
| **TI** | OC | Preclinical | 11811 | <0.0001 | **<0.0001** | -0.11 | -0.15 | -0.081 | -6.57 | 0.32 |
| | Preclinical | Symptomatic | 1180.5 | <0.0001 | **<0.0001** | -0.63 | -0.75 | -0.51 | -7.43 | 0.54 |
| | OC | Symptomatic | 1535.5 | <0.0001 | **<0.0001** | -0.77 | -0.86 | -0.66 | -9.44 | 0.51 |
| **TSS** | OC | Preclinical | 13659.5 | <0.0001 | **<0.0001** | -0.014 | -0.023 | -0.0073 | -5.00 | 0.24 |
| | Preclinical | Symptomatic | 1361.5 | <0.0001 | **<0.0001** | -0.16 | -0.23 | -0.12 | -6.91 | 0.50 |
| | OC | Symptomatic | 1680.5 | <0.0001 | **<0.0001** | -0.19 | -0.26 | -0.15 | -9.23 | 0.50 |

Comparison between disease-stage groups for each tau metric. Pairwise comparisons conducted with Wilcoxon-Mann-Whitney U tests. P-values reported before (raw) and after (adjusted) multiple comparison correction using Benjamini-Hochberg procedure.

**Supplemental Table 3. Cognitive Composites vs. Tau Metrics per AD Disease Stage.**

| | | Older Controls | | | Preclinical | | | Symptomatic | | |
|---|---|---|---|---|---|---|---|---|---|---|
| | | **r** | **p (raw)** | **p (adjusted)** | **r** | **p (raw)** | **p (adjusted)** | **r** | **p (raw)** | **p (adjusted)** |
| **PACC** | **TI** | 0.014 | 0.82 | 0.82 | -0.17 | 0.041 | 0.062 | -0.30 | 0.030 | 0.061 |
| | **TSS** | -0.090 | 0.13 | 0.13 | -0.27 | 0.0016 | **0.0047** | -0.37 | 0.0071 | **0.011** |
| **Episodic** | **TI** | 0.029 | 0.70 | 0.94 | 0.0085 | 0.94 | 0.94 | -0.37 | 0.0058 | **0.017** |
| | **TSS** | -0.15 | 0.044 | 0.067 | -0.15 | 0.17 | 0.17 | -0.33 | 0.015 | **0.044** |
| **Semantic** | **TI** | 0.0077 | 0.92 | 0.92 | -0.034 | 0.75 | 0.92 | -0.36 | 0.0066 | **0.020** |
| | **TSS** | -0.038 | 0.61 | 0.61 | -0.17 | 0.098 | 0.15 | -0.38 | 0.0050 | **0.015** |
| **Working** | **TI** | 0.014 | 0.85 | 0.85 | 0.074 | 0.49 | 0.85 | -0.029 | 0.84 | 0.85 |
| | **TSS** | 0.018 | 0.81 | 0.81 | -0.076 | 0.47 | 0.71 | -0.18 | 0.18 | 0.55 |
| **Attention** | **TI** | -0.041 | 0.50 | 0.50 | -0.21 | 0.012 | **0.037** | -0.29 | 0.034 | 0.050 |
| | **TSS** | -0.13 | 0.030 | **0.030** | -0.27 | 0.0013 | **0.0020** | -0.44 | 0.0010 | **0.0020** |

Spearman correlation calculated for each disease stage group between cognitive composite scores and tau metrics. P-values reported before (raw) and after (adjusted) multiple comparison correction using Benjamini-Hochberg procedure.

**Supplemental Table 4. Baseline Cognition Regression Coefficients.**

Linear regressions were fit to participant data estimating baseline cognitive composite score from baseline tau metrics and covariates in four semi-nested models. Beta coefficient reported for each model variable with 95% confidence interval (CI) and significance evaluated with t-statistic.

**Baseline - Knight PACC**

| | Covariate Model | | | | TI Model | | | | TSS Model | | | | Additive Model | | | |
|---|---|---|---|---|---|---|---|---|---|---|---|---|---|---|---|---|
| *Predictors* | *Beta* | *95% CI* | *t* | *p* | *Beta* | *95% CI* | *t* | *p* | *Beta* | *95% CI* | *t* | *p* | *Beta* | *95% CI* | *t* | *p* |
| Intercept | 0.52 | -0.41 – 1.46 | 1.10 | 0.2708 | 1.52 | 0.68 – 2.37 | 3.53 | **0.0005** | 0.46 | -0.37 – 1.28 | 1.09 | 0.2774 | 0.98 | 0.10 – 1.86 | 2.19 | **0.0290** |
| Age | -0.04 | -0.05 – -0.03 | -7.55 | **<0.0001** | -0.03 | -0.03 – -0.02 | -5.61 | **<0.0001** | -0.03 | -0.04 – -0.02 | -6.69 | **<0.0001** | -0.03 | -0.04 – -0.02 | -5.98 | **<0.0001** |
| Sex (F) | -0.20 | -0.28 – -0.12 | -5.01 | **<0.0001** | -0.24 | -0.31 – -0.17 | -6.68 | **<0.0001** | -0.19 | -0.26 – -0.12 | -5.44 | **<0.0001** | -0.21 | -0.28 – -0.14 | -6.00 | **<0.0001** |
| Education | 0.12 | 0.08 – 0.15 | 6.79 | **<0.0001** | 0.10 | 0.07 – 0.13 | 6.47 | **<0.0001** | 0.10 | 0.07 – 0.13 | 6.67 | **<0.0001** | 0.10 | 0.07 – 0.13 | 6.55 | **<0.0001** |
| TI | | | | | -1.22 | -1.43 – -1.00 | -11.20 | **<0.0001** | | | | | -0.61 | -0.99 – -0.23 | -3.13 | **0.0018** |
| TSS | | | | | | | | | -3.32 | -3.89 – -2.75 | -11.44 | **<0.0001** | -1.96 | -2.98 – -0.94 | -3.77 | **0.0002** |
| Observations | 471 | | | | 471 | | | | 471 | | | | 471 | | | |
| $R^2$ / $R^2$ adjusted | 0.231 / 0.226 | | | | 0.394 / 0.389 | | | | 0.399 / 0.394 | | | | 0.412 / 0.405 | | | |

**Baseline - Episodic Memory**

| | Covariate Model | | | | TI Model | | | | TSS Model | | | | Additive Model | | | |
|---|---|---|---|---|---|---|---|---|---|---|---|---|---|---|---|---|
| *Predictors* | *Beta* | *95% CI* | *t* | *p* | *Beta* | *95% CI* | *t* | *p* | *Beta* | *95% CI* | *t* | *p* | *Beta* | *95% CI* | *t* | *p* |
| Intercept | -1.39 | -2.98 – 0.19 | -1.73 | 0.0840 | 0.65 | -0.74 – 2.04 | 0.92 | 0.3606 | -0.55 | -1.93 – 0.82 | -0.79 | 0.4299 | 0.32 | -1.09 – 1.72 | 0.44 | 0.6579 |
| Age | -0.01 | -0.03 – 0.01 | -1.00 | 0.3171 | -0.00 | -0.02 – 0.01 | -0.26 | 0.7966 | -0.01 | -0.02 – 0.00 | -1.31 | 0.1910 | -0.00 | -0.02 – 0.01 | -0.62 | 0.5335 |
| Sex (F) | -0.21 | -0.33 – -0.10 | -3.64 | **0.0003** | -0.25 | -0.35 – -0.16 | -5.12 | **<0.0001** | -0.19 | -0.29 – -0.09 | -3.77 | **0.0002** | -0.23 | -0.33 – -0.13 | -4.61 | **<0.0001** |
| Education | 0.10 | 0.05 – 0.15 | 4.07 | **0.0001** | 0.07 | 0.03 – 0.12 | 3.37 | **0.0008** | 0.08 | 0.03 – 0.12 | 3.52 | **0.0005** | 0.07 | 0.03 – 0.11 | 3.37 | **0.0008** |
| TI | | | | | -1.54 | -1.80 – -1.27 | -11.25 | **<0.0001** | | | | | -1.02 | -1.52 – -0.53 | -4.10 | **0.0001** |
| TSS | | | | | | | | | -3.95 | -4.68 – -3.21 | -10.59 | **<0.0001** | -1.63 | -2.95 – -0.31 | -2.43 | **0.0156** |
| Observations | 328 | | | | 328 | | | | 328 | | | | 328 | | | |
| $R^2$ / $R^2$ adjusted | 0.081 / 0.073 | | | | 0.340 / 0.332 | | | | 0.318 / 0.310 | | | | 0.352 / 0.342 | | | |

**Baseline - Semantic Memory**

| | Covariate Model | | | | TI Model | | | | TSS Model | | | | Additive Model | | | |
|---|---|---|---|---|---|---|---|---|---|---|---|---|---|---|---|---|
| *Predictors* | *Beta* | *95% CI* | *t* | *p* | *Beta* | *95% CI* | *t* | *p* | *Beta* | *95% CI* | *t* | *p* | *Beta* | *95% CI* | *t* | *p* |
| Intercept | -0.88 | -2.22 – 0.46 | -1.29 | 0.1985 | 0.67 | -0.56 – 1.90 | 1.08 | 0.2831 | -0.21 | -1.42 – 0.99 | -0.35 | 0.7261 | 0.39 | -0.85 – 1.64 | 0.62 | 0.5370 |
| Age | -0.02 | -0.04 – -0.01 | -2.99 | **0.0030** | -0.02 | -0.03 – -0.00 | -2.51 | **0.0126** | -0.02 | -0.03 – -0.01 | -3.35 | **0.0009** | -0.02 | -0.03 – -0.01 | -2.83 | **0.0050** |
| Sex (F) | -0.16 | -0.26 – -0.06 | -3.23 | **0.0014** | -0.19 | -0.28 – -0.10 | -4.30 | **<0.0001** | -0.14 | -0.23 – -0.05 | -3.15 | **0.0018** | -0.17 | -0.26 – -0.08 | -3.78 | **0.0002** |
| Education | 0.13 | 0.09 – 0.18 | 6.29 | **<0.0001** | 0.11 | 0.07 – 0.14 | 5.59 | **<0.0001** | 0.11 | 0.07 – 0.15 | 5.71 | **<0.0001** | 0.11 | 0.07 – 0.14 | 5.57 | **<0.0001** |
| TI | | | | | -1.12 | -1.36 – -0.89 | -9.55 | **<0.0001** | | | | | -0.70 | -1.12 – -0.27 | -3.23 | **0.0014** |
| TSS | | | | | | | | | -2.95 | -3.58 – -2.32 | -9.23 | **<0.0001** | -1.37 | -2.52 – -0.23 | -2.37 | **0.0186** |
| Observations | 332 | | | | 332 | | | | 332 | | | | 332 | | | |
| $R^2$ / $R^2$ adjusted | 0.158 / 0.150 | | | | 0.342 / 0.334 | | | | 0.332 / 0.324 | | | | 0.353 / 0.343 | | | |

**Baseline - Working Memory**

| | Covariate Model | | | | TI Model | | | | TSS Model | | | | Additive Model | | | |
|---|---|---|---|---|---|---|---|---|---|---|---|---|---|---|---|---|
| *Predictors* | *Beta* | *95% CI* | *t* | *p* | *Beta* | *95% CI* | *t* | *p* | *Beta* | *95% CI* | *t* | *p* | *Beta* | *95% CI* | *t* | *p* |
| Intercept | -0.76 | -2.00 – 0.48 | -1.20 | 0.2292 | -0.08 | -1.33 – 1.17 | -0.13 | 0.8996 | -0.41 | -1.62 – 0.80 | -0.67 | 0.5037 | -0.38 | -1.65 – 0.88 | -0.59 | 0.5533 |
| Age | -0.02 | -0.03 – -0.00 | -2.29 | **0.0229** | -0.01 | -0.03 – 0.00 | -1.96 | 0.0504 | -0.02 | -0.03 – -0.00 | -2.36 | **0.0187** | -0.02 | -0.03 – -0.00 | -2.30 | **0.0219** |
| Sex (F) | -0.02 | -0.11 – 0.07 | -0.54 | 0.5917 | -0.04 | -0.13 – 0.05 | -0.83 | 0.4048 | -0.01 | -0.10 – 0.07 | -0.31 | 0.7567 | -0.02 | -0.10 – 0.07 | -0.33 | 0.7387 |
| Education | 0.10 | 0.06 – 0.14 | 5.23 | **<0.0001** | 0.09 | 0.05 – 0.13 | 4.68 | **<0.0001** | 0.09 | 0.05 – 0.13 | 4.69 | **<0.0001** | 0.09 | 0.05 – 0.13 | 4.66 | **<0.0001** |
| TI | | | | | -0.49 | -0.73 – -0.26 | -4.10 | **0.0001** | | | | | -0.03 | -0.47 – 0.40 | -0.15 | 0.8797 |
| TSS | | | | | | | | | -1.55 | -2.18 – -0.92 | -4.83 | **<0.0001** | -1.47 | -2.64 – -0.31 | -2.49 | **0.0133** |
| Observations | 332 | | | | 332 | | | | 332 | | | | 332 | | | |
| $R^2$ / $R^2$ adjusted | 0.101 / 0.093 | | | | 0.145 / 0.135 | | | | 0.161 / 0.151 | | | | 0.161 / 0.149 | | | |

**Baseline - Attention/Processing**

| | Covariate Model | | | | TI Model | | | | TSS Model | | | | Additive Model | | | |
|---|---|---|---|---|---|---|---|---|---|---|---|---|---|---|---|---|
| *Predictors* | *Beta* | *95% CI* | *t* | *p* | *Beta* | *95% CI* | *t* | *p* | *Beta* | *95% CI* | *t* | *p* | *Beta* | *95% CI* | *t* | *p* |
| Intercept | 0.27 | -0.89 – 1.43 | 0.45 | 0.6497 | 1.49 | 0.43 – 2.54 | 2.77 | **0.0058** | 0.19 | -0.82 – 1.20 | 0.37 | 0.7123 | 0.66 | -0.42 – 1.74 | 1.20 | 0.2322 |
| Age | -0.04 | -0.05 – -0.03 | -6.41 | **<0.0001** | -0.02 | -0.04 – -0.01 | -4.32 | **<0.0001** | -0.03 | -0.04 – -0.02 | -5.32 | **<0.0001** | -0.03 | -0.04 – -0.02 | -4.78 | **<0.0001** |
| Sex (F) | -0.16 | -0.26 – -0.07 | -3.34 | **0.0009** | -0.21 | -0.30 – -0.12 | -4.76 | **<0.0001** | -0.15 | -0.24 – -0.07 | -3.50 | **0.0005** | -0.17 | -0.26 – -0.08 | -3.91 | **0.0001** |
| Education | 0.14 | 0.09 – 0.18 | 6.39 | **<0.0001** | 0.11 | 0.08 – 0.15 | 5.98 | **<0.0001** | 0.11 | 0.08 – 0.15 | 6.12 | **<0.0001** | 0.11 | 0.08 – 0.15 | 6.04 | **<0.0001** |
| TI | | | | | -1.48 | -1.75 – -1.22 | -11.09 | **<0.0001** | | | | | -0.54 | -1.01 – -0.08 | -2.31 | **0.0211** |
| TSS | | | | | | | | | -4.20 | -4.88 – -3.51 | -12.05 | **<0.0001** | -3.00 | -4.22 – -1.77 | -4.80 | **<0.0001** |
| Observations | 466 | | | | 466 | | | | 466 | | | | 466 | | | |
| $R^2$ / $R^2$ adjusted | 0.184 / 0.179 | | | | 0.356 / 0.350 | | | | 0.379 / 0.374 | | | | 0.386 / 0.380 | | | |

**Supplemental Table 5. Longitudinal Cognition Regression Coefficients.**

Linear mixed effects regressions were fit to participant data estimating longitudinal cognitive composite score from baseline tau metrics and covariates in four semi-nested models. Beta coefficient reported for each model variable with 95% confidence interval (CI) and significance evaluated with t-statistic.

**Longitudinal - Knight PACC**

| | Covariate Model | | | | TI Model | | | | TSS Model | | | | Additive Model | | | |
|---|---|---|---|---|---|---|---|---|---|---|---|---|---|---|---|---|
| *Predictors* | *Beta* | *95% CI* | *t* | *p* | *Beta* | *95% CI* | *t* | *p* | *Beta* | *95% CI* | *t* | *p* | *Beta* | *95% CI* | *t* | *p* |
| Intercept | 0.92 | -0.17 – 2.01 | 1.66 | 0.0970 | 2.00 | 1.00 – 3.00 | 3.93 | **0.0001** | 0.98 | -0.01 – 1.96 | 1.95 | 0.0516 | 1.64 | 0.60 – 2.69 | 3.08 | **0.0021** |
| Time (years) | 0.40 | 0.19 – 0.61 | 3.70 | **0.0002** | 0.50 | 0.30 – 0.70 | 4.83 | **<0.0001** | 0.34 | 0.13 – 0.54 | 3.22 | **0.0013** | 0.52 | 0.30 – 0.74 | 4.70 | **<0.0001** |
| Age [BL] | -0.03 | -0.04 – -0.02 | -5.32 | **<0.0001** | -0.02 | -0.04 – -0.01 | -4.49 | **<0.0001** | -0.03 | -0.04 – -0.02 | -5.14 | **<0.0001** | -0.03 | -0.04 – -0.01 | -4.67 | **<0.0001** |
| Sex (F) [BL] | 0.19 | 0.11 – 0.27 | 4.57 | **<0.0001** | 0.26 | 0.19 – 0.34 | 6.84 | **<0.0001** | 0.22 | 0.15 – 0.30 | 5.86 | **<0.0001** | 0.25 | 0.18 – 0.33 | 6.51 | **<0.0001** |
| Education [BL] | 0.07 | 0.04 – 0.11 | 4.01 | **0.0001** | 0.06 | 0.03 – 0.10 | 3.81 | **0.0001** | 0.06 | 0.03 – 0.10 | 3.89 | **0.0001** | 0.06 | 0.03 – 0.09 | 3.81 | **0.0001** |
| Time * Age | -0.01 | -0.01 – -0.00 | -5.72 | **<0.0001** | -0.00 | -0.01 – -0.00 | -3.97 | **0.0001** | -0.01 | -0.01 – -0.00 | -4.79 | **<0.0001** | -0.00 | -0.01 – -0.00 | -3.95 | **0.0001** |
| Time * Sex | -0.01 | -0.02 – 0.01 | -0.72 | 0.4694 | 0.01 | -0.01 – 0.02 | 0.82 | 0.4113 | -0.00 | -0.02 – 0.01 | -0.29 | 0.7748 | 0.01 | -0.01 – 0.02 | 0.90 | 0.3659 |
| Time * Education | 0.00 | -0.01 – 0.01 | 0.16 | 0.8742 | 0.00 | -0.01 – 0.01 | 0.41 | 0.6810 | 0.00 | -0.01 – 0.01 | 0.18 | 0.8559 | 0.00 | -0.00 – 0.01 | 0.43 | 0.6638 |
| TI [BL] | | | | | -1.13 | -1.37 – -0.89 | -9.17 | **<0.0001** | | | | | -0.72 | -1.17 – -0.28 | -3.23 | **0.0013** |
| Time * TI | | | | | -0.22 | -0.28 – -0.15 | -6.63 | **<0.0001** | | | | | -0.24 | -0.35 – -0.14 | -4.50 | **<0.0001** |
| TSS [BL] | | | | | | | | | -3.06 | -3.74 – -2.37 | -8.76 | **<0.0001** | -1.36 | -2.59 – -0.13 | -2.16 | **0.0309** |
| Time * TSS | | | | | | | | | -0.47 | -0.66 – -0.28 | -4.84 | **<0.0001** | 0.09 | -0.22 – 0.40 | 0.58 | 0.5620 |
| **Random Effects** | | | | | | | | | | | | | | | | |
| $\sigma^2$ | 0.10 | | | | 0.10 | | | | 0.10 | | | | 0.10 | | | |
| $\tau_{00}$ | 0.58 Subject | | | | 0.45 Subject | | | | 0.46 Subject | | | | 0.44 Subject | | | |
| $\tau_{11}$ | 0.01 Subject.TIME | | | | 0.01 Subject.TIME | | | | 0.01 Subject.TIME | | | | 0.01 Subject.TIME | | | |
| $\rho_{01}$ | 0.42 Subject | | | | 0.26 Subject | | | | 0.31 Subject | | | | 0.27 Subject | | | |
| ICC | 0.88 | | | | 0.85 | | | | 0.85 | | | | 0.85 | | | |
| N | 371 Subject | | | | 371 Subject | | | | 371 Subject | | | | 371 Subject | | | |
| Observations | 1239 | | | | 1239 | | | | 1239 | | | | 1239 | | | |
| Marginal $R^2$ / Conditional $R^2$ | 0.175 / 0.903 | | | | 0.334 / 0.898 | | | | 0.304 / 0.897 | | | | 0.336 / 0.897 | | | |

**Longitudinal - Episodic Memory**

| | **Covariate Model** | | | | **TI Model** | | | | **TSS Model** | | | | **Additive Model** | | | |
|---|---|---|---|---|---|---|---|---|---|---|---|---|---|---|---|---|
| *Predictors* | *Beta* | *95% CI* | *t* | *p* | *Beta* | *95% CI* | *t* | *p* | *Beta* | *95% CI* | *t* | *p* | *Beta* | *95% CI* | *t* | *p* |
| Intercept | -0.59 | -2.15 – 0.97 | -0.74 | 0.4584 | 1.04 | -0.42 – 2.49 | 1.40 | 0.1620 | -0.43 | -1.88 – 1.02 | -0.58 | 0.5633 | 0.92 | -0.58 – 2.42 | 1.20 | 0.2309 |
| Time (years) | 0.74 | 0.43 – 1.05 | 4.68 | **<0.0001** | 0.95 | 0.66 – 1.25 | 6.29 | **<0.0001** | 0.70 | 0.39 – 1.00 | 4.44 | **<0.0001** | 1.06 | 0.76 – 1.36 | 6.91 | **<0.0001** |
| Age [BL] | -0.01 | -0.03 – 0.00 | -1.40 | 0.1616 | -0.01 | -0.02 – 0.01 | -0.70 | 0.4828 | -0.01 | -0.03 – 0.01 | -1.14 | 0.2551 | -0.01 | -0.02 – 0.01 | -0.80 | 0.4210 |
| Sex (F) [BL] | 0.25 | 0.14 – 0.35 | 4.66 | **<0.0001** | 0.30 | 0.21 – 0.40 | 6.41 | **<0.0001** | 0.26 | 0.16 – 0.35 | 5.31 | **<0.0001** | 0.30 | 0.21 – 0.39 | 6.29 | **<0.0001** |
| Education [BL] | 0.08 | 0.03 – 0.12 | 3.32 | **0.0009** | 0.06 | 0.02 – 0.10 | 2.87 | **0.0042** | 0.07 | 0.03 – 0.11 | 3.22 | **0.0013** | 0.06 | 0.02 – 0.10 | 2.91 | **0.0037** |
| Time * Age | -0.01 | -0.01 – -0.01 | -5.13 | **<0.0001** | -0.01 | -0.01 – -0.00 | -4.15 | **<0.0001** | -0.01 | -0.01 – -0.00 | -4.63 | **<0.0001** | -0.01 | -0.01 – -0.00 | -4.05 | **0.0001** |
| Time * Sex | -0.02 | -0.04 – 0.00 | -1.90 | 0.0577 | -0.00 | -0.02 – 0.01 | -0.49 | 0.6238 | -0.02 | -0.03 – 0.00 | -1.60 | 0.1108 | -0.00 | -0.02 – 0.02 | -0.22 | 0.8242 |
| Time * Education | -0.00 | -0.01 – 0.00 | -1.01 | 0.3120 | -0.00 | -0.01 – 0.00 | -1.26 | 0.2073 | -0.00 | -0.01 – 0.00 | -1.06 | 0.2886 | -0.01 | -0.01 – 0.00 | -1.32 | 0.1887 |
| TI [BL] | | | | | -1.42 | -1.72 – -1.13 | -9.48 | **<0.0001** | | | | | -1.26 | -1.76 – -0.76 | -4.95 | **<0.0001** |
| Time * TI | | | | | -0.29 | -0.36 – -0.22 | -7.82 | **<0.0001** | | | | | -0.40 | -0.51 – -0.30 | -7.49 | **<0.0001** |
| TSS [BL] | | | | | | | | | -3.74 | -4.64 – -2.85 | -8.21 | **<0.0001** | -0.60 | -2.07 – 0.87 | -0.80 | 0.4243 |
| Time * TSS | | | | | | | | | -0.38 | -0.59 – -0.18 | -3.65 | **0.0003** | 0.42 | 0.14 – 0.71 | 2.95 | **0.0033** |
| **Random Effects** | | | | | | | | | | | | | | | | |
| $\sigma^2$ | 0.15 | | | | 0.14 | | | | 0.14 | | | | 0.14 | | | |
| $\tau_{00}$ | 0.84 Subject | | | | 0.64 Subject | | | | 0.69 Subject | | | | 0.64 Subject | | | |
| $\tau_{11}$ | 0.02 Subject.TIME | | | | 0.01 Subject.TIME | | | | 0.02 Subject.TIME | | | | 0.01 Subject.TIME | | | |
| $\rho_{01}$ | 0.09 Subject | | | | -0.20 Subject | | | | -0.03 Subject | | | | -0.17 Subject | | | |
| ICC | 0.88 | | | | 0.83 | | | | 0.85 | | | | 0.83 | | | |
| N | 373 Subject | | | | 373 Subject | | | | 373 Subject | | | | 373 Subject | | | |
| Observations | 1560 | | | | 1560 | | | | 1560 | | | | 1560 | | | |
| Marginal $R^2$ / Conditional $R^2$ | 0.093 / 0.892 | | | | 0.325 / 0.882 | | | | 0.242 / 0.890 | | | | 0.325 / 0.882 | | | |

**Longitudinal - Semantic Memory**

| | **Covariate Model** | | | | **TI Model** | | | | **TSS Model** | | | | **Additive Model** | | | |
|---|---|---|---|---|---|---|---|---|---|---|---|---|---|---|---|---|
| *Predictors* | *Beta* | *95% CI* | *t* | *p* | *Beta* | *95% CI* | *t* | *p* | *Beta* | *95% CI* | *t* | *p* | *Beta* | *95% CI* | *t* | *p* |
| Intercept | -1.33 | -2.61 – -0.05 | -2.03 | **0.0424** | 0.57 | -0.66 – 1.79 | 0.91 | 0.3632 | -0.21 | -1.40 – 0.97 | -0.35 | 0.7252 | 0.40 | -0.84 – 1.63 | 0.63 | 0.5287 |
| Time (years) | 0.48 | 0.24 – 0.72 | 3.94 | **0.0001** | 0.77 | 0.53 – 1.00 | 6.45 | **<0.0001** | 0.50 | 0.26 – 0.74 | 4.15 | **<0.0001** | 0.79 | 0.55 – 1.02 | 6.45 | **<0.0001** |
| Age [BL] | -0.01 | -0.02 – 0.00 | -1.44 | 0.1505 | -0.01 | -0.03 – 0.00 | -1.94 | 0.0529 | -0.02 | -0.03 – -0.00 | -2.66 | **0.0079** | -0.01 | -0.03 – -0.00 | -2.27 | **0.0236** |
| Sex (F) [BL] | 0.12 | 0.03 – 0.20 | 2.53 | **0.0116** | 0.17 | 0.09 – 0.25 | 4.13 | **<0.0001** | 0.13 | 0.05 – 0.21 | 3.16 | **0.0016** | 0.16 | 0.08 – 0.24 | 3.81 | **0.0001** |
| Education [BL] | 0.12 | 0.08 – 0.15 | 5.92 | **<0.0001** | 0.09 | 0.06 – 0.13 | 5.25 | **<0.0001** | 0.09 | 0.06 – 0.13 | 5.23 | **<0.0001** | 0.09 | 0.06 – 0.12 | 5.21 | **<0.0001** |
| Time * Age | -0.01 | -0.01 – -0.00 | -5.62 | **<0.0001** | -0.01 | -0.01 – -0.00 | -5.15 | **<0.0001** | -0.01 | -0.01 – -0.00 | -5.42 | **<0.0001** | -0.01 | -0.01 – -0.00 | -4.92 | **<0.0001** |
| Time * Sex | 0.01 | -0.01 – 0.02 | 0.75 | 0.4507 | 0.02 | 0.00 – 0.03 | 2.65 | **0.0081** | 0.01 | -0.00 – 0.02 | 1.31 | 0.1903 | 0.02 | 0.01 – 0.03 | 2.75 | **0.0060** |
| Time * Education | 0.00 | -0.01 – 0.01 | 0.30 | 0.7665 | -0.00 | -0.01 – 0.01 | -0.32 | 0.7456 | -0.00 | -0.01 – 0.01 | -0.03 | 0.9762 | -0.00 | -0.01 – 0.01 | -0.30 | 0.7660 |
| TI [BL] | | | | | -1.03 | -1.25 – -0.81 | -9.29 | **<0.0001** | | | | | -0.69 | -1.10 – -0.28 | -3.33 | **0.0009** |
| Time * TI | | | | | -0.27 | -0.32 – -0.21 | -9.54 | **<0.0001** | | | | | -0.30 | -0.39 – -0.21 | -6.90 | **<0.0001** |
| TSS [BL] | | | | | | | | | -2.76 | -3.35 – -2.16 | -9.11 | **<0.0001** | -1.08 | -2.19 – 0.03 | -1.91 | 0.0566 |
| Time * TSS | | | | | | | | | -0.51 | -0.66 – -0.36 | -6.78 | **<0.0001** | 0.11 | -0.11 – 0.33 | 1.01 | 0.3110 |
| **Random Effects** | | | | | | | | | | | | | | | | |
| $\sigma^2$ | 0.16 | | | | 0.15 | | | | 0.15 | | | | 0.15 | | | |
| $\tau_{00}$ | 0.63 Subject | | | | 0.47 Subject | | | | 0.48 Subject | | | | 0.47 Subject | | | |
| $\tau_{11}$ | 0.01 Subject.TIME | | | | 0.00 Subject.TIME | | | | 0.00 Subject.TIME | | | | 0.00 Subject.TIME | | | |
| $\rho_{01}$ | 0.46 Subject | | | | 0.14 Subject | | | | 0.29 Subject | | | | 0.15 Subject | | | |
| ICC | 0.84 | | | | 0.78 | | | | 0.80 | | | | 0.78 | | | |
| N | 381 Subject | | | | 381 Subject | | | | 381 Subject | | | | 381 Subject | | | |
| Observations | 1595 | | | | 1595 | | | | 1595 | | | | 1595 | | | |
| Marginal $R^2$ / Conditional $R^2$ | 0.131 / 0.859 | | | | 0.338 / 0.853 | | | | 0.301 / 0.857 | | | | 0.340 / 0.852 | | | |

**Longitudinal - Working Memory**

| | Covariate Model | | | | TI Model | | | | TSS Model | | | | Additive Model | | | |
|---|---|---|---|---|---|---|---|---|---|---|---|---|---|---|---|---|
| *Predictors* | *Beta* | *95% CI* | *t* | *p* | *Beta* | *95% CI* | *t* | *p* | *Beta* | *95% CI* | *t* | *p* | *Beta* | *95% CI* | *t* | *p* |
| Intercept | -0.86 | -2.01 – 0.29 | -1.46 | 0.1434 | -0.12 | -1.31 – 1.07 | -0.19 | 0.8495 | -0.44 | -1.58 – 0.70 | -0.75 | 0.4527 | -0.26 | -1.47 – 0.94 | -0.43 | 0.6686 |
| Time (years) | 0.15 | -0.07 – 0.37 | 1.37 | 0.1716 | 0.24 | 0.01 – 0.46 | 2.03 | **0.0429** | 0.12 | -0.10 – 0.35 | 1.10 | 0.2733 | 0.26 | 0.02 – 0.49 | 2.17 | **0.0305** |
| Age [BL] | -0.01 | -0.02 – 0.00 | -1.40 | 0.1630 | -0.01 | -0.02 – 0.00 | -1.44 | 0.1508 | -0.01 | -0.02 – 0.00 | -1.79 | 0.0734 | -0.01 | -0.02 – 0.00 | -1.65 | 0.0983 |
| Sex (F) [BL] | 0.01 | -0.07 – 0.09 | 0.27 | 0.7867 | 0.03 | -0.04 – 0.11 | 0.84 | 0.3997 | 0.02 | -0.06 – 0.10 | 0.49 | 0.6213 | 0.03 | -0.05 – 0.10 | 0.65 | 0.5162 |
| Education [BL] | 0.08 | 0.05 – 0.12 | 4.79 | **<0.0001** | 0.07 | 0.04 – 0.11 | 4.29 | **<0.0001** | 0.07 | 0.04 – 0.11 | 4.34 | **<0.0001** | 0.07 | 0.04 – 0.11 | 4.29 | **<0.0001** |
| Time * Age | -0.00 | -0.00 – 0.00 | -1.64 | 0.1018 | -0.00 | -0.00 – 0.00 | -1.12 | 0.2619 | -0.00 | -0.00 – 0.00 | -1.28 | 0.2017 | -0.00 | -0.00 – 0.00 | -0.95 | 0.3428 |
| Time * Sex | 0.01 | -0.01 – 0.02 | 0.83 | 0.4045 | 0.01 | -0.00 – 0.02 | 1.52 | 0.1279 | 0.01 | -0.01 – 0.02 | 0.96 | 0.3358 | 0.01 | -0.00 – 0.03 | 1.64 | 0.1018 |
| Time * Education | -0.00 | -0.01 – 0.01 | -0.21 | 0.8353 | -0.00 | -0.01 – 0.01 | -0.23 | 0.8196 | -0.00 | -0.01 – 0.01 | -0.12 | 0.9018 | -0.00 | -0.01 – 0.01 | -0.22 | 0.8238 |
| TI [BL] | | | | | -0.44 | -0.66 – -0.22 | -3.94 | **0.0001** | | | | | -0.20 | -0.60 – 0.21 | -0.94 | 0.3454 |
| Time * TI | | | | | -0.10 | -0.16 – -0.05 | -3.77 | **0.0002** | | | | | -0.14 | -0.22 – -0.06 | -3.27 | **0.0011** |
| TSS [BL] | | | | | | | | | -1.30 | -1.89 – -0.70 | -4.26 | **<0.0001** | -0.79 | -1.91 – 0.32 | -1.39 | 0.1639 |
| Time * TSS | | | | | | | | | -0.15 | -0.29 – -0.01 | -2.11 | **0.0351** | 0.12 | -0.10 – 0.33 | 1.07 | 0.2832 |
| **Random Effects** | | | | | | | | | | | | | | | | |
| $\sigma^2$ | 0.15 | | | | 0.15 | | | | 0.15 | | | | 0.15 | | | |
| $\tau_{00}$ | 0.46 $_{Subject}$ | | | | 0.43 $_{Subject}$ | | | | 0.43 $_{Subject}$ | | | | 0.43 $_{Subject}$ | | | |
| $\tau_{11}$ | 0.00 $_{Subject.TIME}$ | | | | 0.00 $_{Subject.TIME}$ | | | | 0.00 $_{Subject.TIME}$ | | | | 0.00 $_{Subject.TIME}$ | | | |
| $\rho_{01}$ | -0.04 $_{Subject}$ | | | | -0.07 $_{Subject}$ | | | | -0.05 $_{Subject}$ | | | | -0.06 $_{Subject}$ | | | |
| ICC | 0.76 | | | | 0.75 | | | | 0.75 | | | | 0.75 | | | |
| N | 380 $_{Subject}$ | | | | 380 $_{Subject}$ | | | | 380 $_{Subject}$ | | | | 380 $_{Subject}$ | | | |
| Observations | 1592 | | | | 1592 | | | | 1592 | | | | 1592 | | | |
| Marginal $R^2$ / Conditional $R^2$ | 0.072 / 0.781 | | | | 0.122 / 0.782 | | | | 0.116 / 0.782 | | | | 0.124 / 0.783 | | | |

**Longitudinal - Attention/Processing**

| | Covariate Model | | | | TI Model | | | | TSS Model | | | | Additive Model | | | |
|---|---|---|---|---|---|---|---|---|---|---|---|---|---|---|---|---|
| *Predictors* | *Beta* | *95% CI* | *t* | *p* | *Beta* | *95% CI* | *t* | *p* | *Beta* | *95% CI* | *t* | *p* | *Beta* | *95% CI* | *t* | *p* |
| Intercept | 1.19 | -0.01 – 2.40 | 1.94 | 0.0523 | 2.17 | 1.03 – 3.32 | 3.73 | **0.0002** | 1.26 | 0.14 – 2.37 | 2.20 | **0.0277** | 1.75 | 0.56 – 2.95 | 2.87 | **0.0041** |
| Time (years) | 0.08 | -0.20 – 0.36 | 0.58 | 0.5589 | 0.16 | -0.11 – 0.44 | 1.17 | 0.2421 | 0.01 | -0.26 – 0.29 | 0.09 | 0.9284 | 0.16 | -0.14 – 0.45 | 1.03 | 0.3026 |
| Age [BL] | -0.04 | -0.05 – -0.02 | -5.67 | **<0.0001** | -0.03 | -0.04 – -0.02 | -4.93 | **<0.0001** | -0.03 | -0.05 – -0.02 | -5.50 | **<0.0001** | -0.03 | -0.04 – -0.02 | -5.13 | **<0.0001** |
| Sex (F) [BL] | 0.14 | 0.05 – 0.23 | 3.00 | **0.0028** | 0.20 | 0.12 – 0.29 | 4.65 | **<0.0001** | 0.17 | 0.08 – 0.25 | 3.92 | **0.0001** | 0.19 | 0.10 – 0.28 | 4.32 | **<0.0001** |
| Education [BL] | 0.08 | 0.04 – 0.12 | 4.10 | **<0.0001** | 0.07 | 0.04 – 0.11 | 3.91 | **0.0001** | 0.07 | 0.04 – 0.11 | 3.97 | **0.0001** | 0.07 | 0.04 – 0.11 | 3.91 | **0.0001** |
| Time * Age | -0.00 | -0.01 – -0.00 | -2.44 | **0.0149** | -0.00 | -0.00 – 0.00 | -1.18 | 0.2387 | -0.00 | -0.01 – 0.00 | -1.64 | 0.1010 | -0.00 | -0.00 – 0.00 | -1.17 | 0.2427 |
| Time * Sex | -0.00 | -0.02 – 0.02 | -0.20 | 0.8409 | 0.01 | -0.01 – 0.03 | 0.77 | 0.4397 | 0.00 | -0.02 – 0.02 | 0.10 | 0.9237 | 0.01 | -0.01 – 0.03 | 0.75 | 0.4560 |
| Time * Education | 0.01 | -0.00 – 0.01 | 1.24 | 0.2139 | 0.01 | -0.00 – 0.02 | 1.41 | 0.1577 | 0.01 | -0.00 – 0.01 | 1.33 | 0.1844 | 0.01 | -0.00 – 0.02 | 1.42 | 0.1564 |
| TI [BL] | | | | | -1.04 | -1.31 – -0.76 | -7.39 | **<0.0001** | | | | | -0.55 | -1.05 – -0.05 | -2.16 | **0.0308** |
| Time * TI | | | | | -0.19 | -0.27 – -0.10 | -4.29 | **<0.0001** | | | | | -0.18 | -0.32 – -0.04 | -2.52 | **0.0120** |
| TSS [BL] | | | | | | | | | -2.91 | -3.68 – -2.14 | -7.40 | **<0.0001** | -1.62 | -3.02 – -0.21 | -2.26 | **0.0241** |
| Time * TSS | | | | | | | | | -0.45 | -0.70 – -0.20 | -3.50 | **0.0005** | -0.03 | -0.44 – 0.38 | -0.13 | 0.8950 |
| **Random Effects** | | | | | | | | | | | | | | | | |
| $\sigma^2$ | 0.16 | | | | 0.16 | | | | 0.16 | | | | 0.16 | | | |
| $\tau_{00}$ | 0.66 $_{Subject}$ | | | | 0.55 $_{Subject}$ | | | | 0.55 $_{Subject}$ | | | | 0.55 $_{Subject}$ | | | |
| $\tau_{11}$ | 0.01 $_{Subject.TIME}$ | | | | 0.01 $_{Subject.TIME}$ | | | | 0.01 $_{Subject.TIME}$ | | | | 0.01 $_{Subject.TIME}$ | | | |
| $\rho_{01}$ | 0.17 $_{Subject}$ | | | | 0.06 $_{Subject}$ | | | | 0.08 $_{Subject}$ | | | | 0.06 $_{Subject}$ | | | |
| ICC | 0.84 | | | | 0.81 | | | | 0.81 | | | | 0.80 | | | |
| N | 367 $_{Subject}$ | | | | 367 $_{Subject}$ | | | | 367 $_{Subject}$ | | | | 367 $_{Subject}$ | | | |
| Observations | 1228 | | | | 1228 | | | | 1228 | | | | 1228 | | | |
| Marginal $R^2$ / Conditional $R^2$ | 0.163 / 0.864 | | | | 0.268 / 0.858 | | | | 0.258 / 0.857 | | | | 0.272 / 0.858 | | | |

**Supplemental Table 6. Participant Longitudinal Clinical Evaluation Survival Counts vs. Baseline Tau Metrics.**

| | | | Year 0 | Year 1 | Year 2 | Year 3 | Year 4 | Year 5 | Year 6 | Year 7 | Year 8 | Year 9 |
|---|---|---|---|---|---|---|---|---|---|---|---|---|
| **TI** | **N at risk** | **Low** | 201 | 197 | 188 | 172 | 130 | 96 | 51 | 22 | 3 | 0 |
| | | **High** | 202 | 195 | 186 | 169 | 130 | 88 | 43 | 21 | 7 | 0 |
| | **N events** | **Low** | 0 | 2 | 3 | 6 | 17 | 19 | 22 | 22 | 22 | 22 |
| | | **High** | 0 | 2 | 7 | 19 | 24 | 32 | 36 | 38 | 38 | 38 |
| | **N censored** | **Low** | 0 | 2 | 10 | 23 | 54 | 86 | 128 | 157 | 176 | 179 |
| | | **High** | 0 | 5 | 9 | 14 | 48 | 82 | 123 | 143 | 158 | 164 |
| **TSS** | **N at risk** | **Low** | 201 | 199 | 190 | 174 | 133 | 100 | 60 | 28 | 4 | 0 |
| | | **High** | 202 | 193 | 184 | 167 | 127 | 84 | 34 | 15 | 6 | 0 |
| | **N events** | **Low** | 0 | 1 | 3 | 7 | 15 | 18 | 21 | 21 | 21 | 21 |
| | | **High** | 0 | 3 | 7 | 18 | 26 | 33 | 37 | 39 | 39 | 39 |
| | **N censored** | **Low** | 0 | 1 | 8 | 20 | 53 | 83 | 120 | 152 | 176 | 180 |
| | | **High** | 0 | 6 | 11 | 17 | 49 | 85 | 131 | 148 | 158 | 163 |

Annual participant summary counts (N, years 0-9) for Kaplan-Meier curve CDR=0 survival analyses based on median split of either TI (cutoff = 1.18) or TSS (cutoff = 0.019). Cumulative number of events (CDR>0) and cumulative number of censored participants (CDR=0, last visit) reported with remaining number of participants at risk (CDR=0, additional follow-up visits).

**Supplemental Table 7. Longitudinal Clinical Evaluation Regression Coefficients.**

Cox proportional hazards regressions were fit to participant data estimating CDR=0 survival from baseline tau metrics and covariates in four semi-nested models. Hazard Ratio (HR) reported for each model variable with 95% confidence interval (CI) and significance evaluated with Wald statistic (z).

**Longitudinal - CDR**

| | **Covariate Model** | | | | **TI Model** | | | | **TSS Model** | | | | **Additive Model** | | | |
|---|---|---|---|---|---|---|---|---|---|---|---|---|---|---|---|---|
| *Predictors* | *HR* | *95% CI* | *z* | *p* | *HR* | *95% CI* | *z* | *p* | *HR* | *95% CI* | *z* | *p* | *HR* | *95% CI* | *z* | *p* |
| Age | 1.11 | 1.07 – 1.15 | 5.55 | **<0.0001** | 1.09 | 1.05 – 1.14 | 4.48 | **<0.0001** | 1.10 | 1.06 – 1.14 | 5.23 | **<0.0001** | 1.09 | 1.05 – 1.14 | 4.45 | **<0.0001** |
| sex [1] | 0.95 | 0.74 – 1.24 | -0.35 | 0.7252 | 0.78 | 0.60 – 1.02 | -1.81 | 0.0706 | 0.92 | 0.70 – 1.19 | -0.65 | 0.5166 | 0.77 | 0.59 – 1.01 | -1.92 | 0.0553 |
| EDUC | 0.98 | 0.88 – 1.09 | -0.37 | 0.7122 | 0.96 | 0.86 – 1.08 | -0.67 | 0.5021 | 0.97 | 0.87 – 1.08 | -0.57 | 0.5660 | 0.96 | 0.86 – 1.08 | -0.65 | 0.5133 |
| TI BL | | | | | 19.85 | 7.25 – 54.31 | 5.82 | **<0.0001** | | | | | 33.73 | 7.44 – 152.90 | 4.56 | **<0.0001** |
| TSS BL | | | | | | | | | 32.84 | 3.65 – 295.40 | 3.12 | **0.0018** | 0.18 | 0.00 – 8.26 | -0.88 | 0.3790 |
| Observations | 403 | | | | 403 | | | | 403 | | | | 403 | | | |
| $R^2$ Nagelkerke | 0.096 | | | | 0.163 | | | | 0.116 | | | | 0.166 | | | |

**Supplemental Table 8. Attention/Processing Speed Model Comparison and Regression Coefficients including Centiloid as a Covariate.**

Comparison of covariate, individual TI, individual TSS, and additive models estimating baseline attention/processing speed composite scores. Linear regressions with covariates age, sex, education, and Centiloid evaluated with log-likelihood and corrected AIC (AICc). Model nominated by AICc (***) and comparable models (*) identified for each cognitive domain composite. Models ordered by AICc with the nominated model listed first. Beta coefficient reported for each model variable with 95% confidence interval (CI) and significance evaluated with t-statistic.

| | Model | K | AICc | Δ AICc | AICc Weight | Log-Likelihood |
|---|---|---|---|---|---|---|
| **PACC** | Additive | 8 | 1250.08 | 0.00 *** | 0.77 | -616.88 |
| | TSS | 7 | 1252.54 | 2.46 | 0.23 | -619.15 |
| | TI | 7 | 1270.50 | 20.42 | 0.00 | -628.13 |
| | Covariate | 6 | 1345.47 | 95.39 | 0.00 | -666.64 |

**Baseline - Attention/Processing (Centiloid)**

| | **Covariate Model** | | | | **TI Model** | | | | **TSS Model** | | | | **Additive Model** | | | |
|---|---|---|---|---|---|---|---|---|---|---|---|---|---|---|---|---|
| *Predictors* | *Beta* | *95% CI* | *t* | *p* | *Beta* | *95% CI* | *t* | *p* | *Beta* | *95% CI* | *t* | *p* | *Beta* | *95% CI* | *t* | *p* |
| Intercept | 0.27 | -0.85 – 1.39 | 0.47 | 0.6381 | 1.44 | 0.38 – 2.51 | 2.67 | **0.0079** | 0.19 | -0.82 – 1.21 | 0.38 | 0.7074 | 0.64 | -0.45 – 1.73 | 1.15 | 0.2499 |
| Age | -0.03 | -0.04 – -0.02 | -4.77 | **<0.0001** | -0.02 | -0.03 – -0.01 | -4.13 | **<0.0001** | -0.03 | -0.04 – -0.02 | -4.95 | **<0.0001** | -0.03 | -0.04 – -0.01 | -4.63 | **<0.0001** |
| Sex (F) | -0.19 | -0.28 – -0.10 | -3.97 | **0.0001** | -0.21 | -0.30 – -0.12 | -4.78 | **<0.0001** | -0.16 | -0.24 – -0.07 | -3.59 | **0.0004** | -0.17 | -0.26 – -0.09 | -3.92 | **0.0001** |
| Education | 0.12 | 0.08 – 0.16 | 5.73 | **<0.0001** | 0.11 | 0.07 – 0.15 | 5.87 | **<0.0001** | 0.11 | 0.08 – 0.15 | 5.98 | **<0.0001** | 0.11 | 0.07 – 0.15 | 5.97 | **<0.0001** |
| Centiloid | -0.02 | -0.03 – -0.01 | -5.87 | **<0.0001** | -0.00 | -0.01 – 0.00 | -0.68 | 0.4960 | -0.00 | -0.01 – 0.00 | -1.00 | 0.3191 | -0.00 | -0.01 – 0.01 | -0.38 | 0.7039 |
| TI | | | | | -1.43 | -1.74 – -1.12 | -9.09 | **<0.0001** | | | | | -0.52 | -1.00 – -0.04 | -2.12 | **0.0347** |
| TSS | | | | | | | | | -4.02 | -4.79 – -3.24 | -10.20 | **<0.0001** | -2.98 | -4.21 – -1.75 | -4.76 | **<0.0001** |
| Observations | 466 | | | | 466 | | | | 466 | | | | 466 | | | |
| $R^2$ / $R^2$ adjusted | 0.241 / 0.234 | | | | 0.356 / 0.349 | | | | 0.381 / 0.374 | | | | 0.387 / 0.379 | | | |
